# Industrial application of heat- and mass balance model for fluid-bed granulation for technology transfer and design space exploration

**DOI:** 10.1016/j.ijpx.2019.100028

**Published:** 2019-08-12

**Authors:** David R. Ochsenbein, Matthew Billups, Bingbing Hong, Elisabeth Schäfer, Alexander J. Marchut, Olav K. Lyngberg

**Affiliations:** a Janssen-Cilag AG, Pharmaceutical Companies of Johnson & Johnson, Switzerland.; b Xian-Janssen Pharmaceutical Ltd., Pharmaceutical Companies of Johnson & Johnson, China; c Janssen Pharmaceutica NV, Pharmaceutical Companies of Johnson & Johnson, Belgium; d Janssen Supply Group, LLC, Pharmaceutical Companies of Johnson & Johnson, United States

**Keywords:** Fluid bed granulation, Technology transfer, Industrial application, Heat- and mass balances, Mechanistic modeling, Digital twin

## Abstract

This work demonstrates the application of state-of-the-art modeling techniques in pharmaceutical manufacturing for fluid bed granulation at varying scales to successfully predict process conditions and ultimately replace experiments during a technology transfer of five products. We describe a mathematical model able to simulate the time-dependent moisture profile in a fluid bed granulation process. The applicability of this model is then demonstrated by calibrating and validating it over a range of operating conditions, manufacturing scales, and formulations. The inherent capability of the moisture profile to serve as a simple, scale-independent surrogate is shown by the large number of successful scale-ups and transfers. A methodology to use this ‘digital twin’ to systematically explore the effects of uncertainty inherent in the process input and model parameter spaces and their impact on the process outputs is described. Two case studies exemplifying the utilization of the model in industrial practice to assess process robustness are provided. Lastly, a pathway to leverage model results to establish proven acceptable ranges for individual parameters is outlined.

## Graphical abstract

**Figure:**
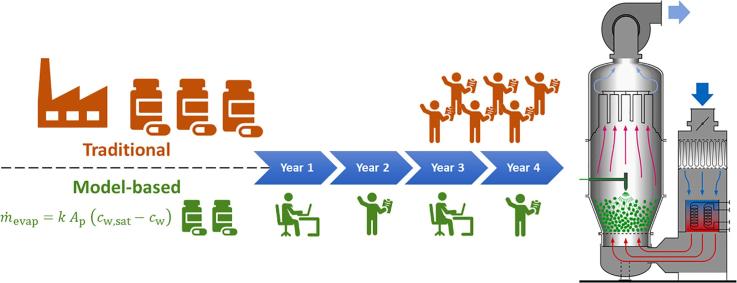

## 1 Introduction

Fluid bed granulation (FBG) is a frequently-used formulation step whose benefits typically include improved flowability and compressibility of powders as well as reducing the risk of segregation. The process consists of two main stages: a spraying phase, during which fluidized particles are coated with an aqueous binder solution and agglomerate, and a drying phase used to evaporate away the water, resulting in a dry powder.

Numerous modeling techniques have been applied to FBG processes. Among the most common approaches are mass-, heat-, and population balance models (PBMs) (Heinrich et al., 2005, Chaudhury et al., 2013, Hu et al., 2008, Muddu et al., 2018, Gupta, 2017), discrete and finite element methods (DEM and FEM), and computational fluid dynamics (CFD) (Sen et al., 2014, Mortier et al., 2011). Modeling studies in literature are mostly concerned with the computation of the evolution of particle size distribution (PSD) as well as granule moisture content typically captured as ‘loss-on-drying’ (LOD), and use granulation time as a process performance metric. PBM, CFD, DEM and FEM are well-suited to describe the evolution of the PSD, yet it remains a formidable task, given that the evolution of the PSD is highly complex and depends on an interplay of effects whose detailed study requires resources in the form of time, equipment, and material. In fact, successfully modeling the PSD can be challenging even in the arguably simpler case of agglomeration during solution crystallization (Ochsenbein, 2015). Heat- and mass balances, on the other hand, are useful to describe the granule moisture trajectories over time as function of process conditions, which include fluidization air flow, inlet air temperature and humidity, binder solution spray rate etc. (Heinrich et al., 2005, Hu et al., 2008, Muddu et al., 2018, Djuris et al., 2017).

While FBG process modeling in academic settings is well-established, its application in the pharmaceutical industry is not. The reasons for this are mostly related to transferability and robustness of models, as will be outlined later. As a consequence, publications of industrially relevant applications of mechanistic models are typically limited in scope (Gupta, 2017, Pauli et al., 2019, Gagnon et al., 2017) and somewhat detailed demonstrations of large-scale successes are exceedingly rare. In the following, we have selected three representative examples (Heinrich et al., 2005, Chaudhury et al., 2013, Hu et al., 2008) for FBG modeling approaches with emphasis on heat and mass balances from literature which will be compared and discussed in detail before introducing key aspects and goals of the alternative approach used in this work.

The approach proposed by Heinrich et al. (2005) is a one-dimensional population balance model which is coupled with the heat and mass transfers in FBG beds to calculate the time-dependent PSD and spatial temperature and humidity distributions. The focus of the study lies on investigating the behavior and phenomena in terms of granule growth, temperature and granule moisture profiles for a continuous fluid bed granulation process. A one-to-one comparison of experimental and calculated results, especially in view of moisture trajectories of the particles, is not a major point of attention. Several aspects should be highlighted with respect to the heat and mass balance modeling part:

- The granulation equipment in the study by Heinrich et al. (2005) is stated to be insulated. Equipment walls and the environment are not part of the heat balances. Consequently, heat loss is not considered in the model.
- A wetting efficiency parameter is applied to describe the ratio of wetted vs. total particle surface and a specific liquid film thickness is assumed.
- The model involves the porosity of the fluidized bed; the authors do not elaborate on how this property is determined.

This approach has been designed and tested for a specific, rather unique continuous granulation set-up. Consequently, the model structure and equations require adaptation and further testing before they can be applied to other FBG processes. For instance, determining values for the wetting efficiency parameter, the thickness of the liquid film around the particles, and the porosity of the fluidized bed may be challenging if these parameters are not treated as fitting parameters.

In the study by Chaudhury et al. (2013) the authors combine a heat- and mass balance model with a three-dimensional PBM. The 3D PBM with a mechanistic aggregation kernel considers granule size, binder content and porosity as process characteristics. In that work, the emphasis lies on gaining deeper insights into the mechanisms of granulation dynamics with respect to particle diameter, moisture content and temperature evolution at different granulation conditions. Furthermore, the model *itself* is being scrutinized, delivering a proof of concept with limited comparison between experimental and calculated results, although the authors compare the general trends of their modeling approach to experimental data by Hu et al., 2008, Rambali et al., 2001. Simulation of granulation scenarios is only done for runs at 2 kg scale with varying fluidization air flow, binder spray rate, inlet air temperature and humidity conditions, without further specifying the granulation equipment.

In relation to our work some relevant aspects of the heat- and mass balance model by Chaudhury et al. (2013) include:

- An experimental correction factor, γ, is introduced in the equation describing the evaporation of moisture. In the expression to calculate the wet surface area as fraction of the total particle surface area, the authors further apply a factor, η, which is not further explained.
- The heat balance is reduced to the temperature calculation of the outlet air and the bed. The authors account for heat loss through an empirical correction quantity.

Chaudhury et al. (2013) do not discuss how they obtained values for factors γ and η, nor how they determined the heat loss quantity. To further generalize the model, a workflow would need to be established that defines how values for the empirical parameters are determined. Furthermore, the predictive capability of the model would need to be investigated in depth by assessing whether it is also applicable under different granulation conditions.

The fluid bed granulation model proposed by Hu et al. (2008) is solely concerned with predicting and controlling the moisture content of the particles during granulation to achieve desired granule properties. The study evaluates the effect of binder spray rate, binder solution concentration, fluidization air flow, inlet air temperature and humidity on the granule humidity. The model is tested against various experimental datasets generated for fluid bed granulations at different scale, i.e., different Glatt granulators (GPCG-1, GPCG-15, GPCG-60). Hu et al. demonstrate how the proposed model can be used to determine appropriate granulation conditions to achieve desired granule moisture trajectories. Some of the specific features of the moisture balance modeling approach suggested are:

- It is thought that two important assumptions hold, namely a) that moisture evaporation happens only after contact of the spray droplets with the powder, and b) that the moisture content of the bed is uniform, i.e., there are no moisture gradients in the bed. Both assumptions appear reasonable considering that in a well-designed FBG process an appropriate combination of powder fluidization and binder spray is applied. In fact, well-mixedness of the powder bed is a prerequisite for a proper process.
- In the expression to calculate the moisture evaporation, the authors incorporate a formulation specific correction factor α that is employed to represent – in a lumped manner – several, difficult-to-determine parameters such as the wetting surface area of the particle bed. In the examples Hu et al. (2008) evaluate in their work, α is determined once with granulation data generated on GPCG-1 equipment and then used for all scales, including for granulations on GPCG-15 and GPCG-60 of the same formulation.
- Notably, the approach comprises only the granule moisture balance, that is, a heat balance to calculate, e.g., the bed temperature is not considered. Consequently, the model uses the inlet air temperature to solve the equation for moisture evaporation. This underlying assumption does not seem to agree with practical FBG experience, where the bed temperature is commonly approaching the wet bulb temperature during spraying and therefore considerably colder than the inlet air.

Overall, the authors present a viable fit-for-purpose approach focusing on the practical engineering task to define appropriate FBG conditions based on achieving a certain moisture trajectory of the granules. In their study, the approach is tested only for one formulation. While a closer look at the comparison between experimental and calculated moisture trajectories reveals certain shortcomings, e.g., with respect to accurately representing the effect of different spray rate conditions and inlet air temperatures, the authors clearly demonstrate how a comparably simple modeling approach can very well support developing an FBG process in a more scientific and lean manner.

Bringing the three examples by Heinrich et al., 2005, Chaudhury et al., 2013, Hu et al., 2008 into context with our work three major aspects should be addressed:

First, in full alignment with Hu et al. we desire a modeling approach that is sufficiently scientifically detailed, yet as simple as possible, to guide us in a reliable way to define appropriate FBG conditions with minimal consumption of resources. Specific challenges we face are mainly related to a wide range of formulations in combination with a diverse landscape of FBG equipment and scales. In addition, we are applying the modeling approach in an industrial environment where time and detailed experimental data is often scarce. Data generated at small scale should ideally enable a fully predictable process transfer to full scale equipment. Any special requirements for data generation that is not related to compliance directly poses a challenge.

Second, the prediction of moisture and temperature trajectories can be sufficient to arrive at a highly practically useful modeling tool that can guide us towards appropriate FBG conditions in a lean manner. While PBMs such as those presented by Heinrich et al., 2005, Chaudhury et al., 2013 are theoretically attractive, to our knowledge no literature work has provided evidence that these can be trained in one granulator and then used directly in another granulator of similar or different size to predict product quality. We further note that measurements for PSD of fluid bed granulated materials are notoriously difficult to interpret, regardless of the method used (de Albuquerque et al., 2016). Even after considering particle segregation and therefore the effects of sampling location on measured PSD, or granule friability, it has been our empirical observation that different processes can result in powders that have a similar distribution yet behave somewhat differently during subsequent compression steps. The inverse may also be true: in our experience granule distributions that ostensibly are very different can at times result in very similarly behaving compression and final product outcomes. In addition to the potential problems that make it difficult to obtain a representative PSD mentioned above, we attribute these observations to the standardization effect of intermediate milling steps, to the complex interaction of solids in systems with wide PSDs, and generally to the large number of other factors that influence final dissolution properties, potentially masking the effect of particle size.

Third, in contrast to other works in literature, high accuracy of the predictions is of major importance to us. Trends alone may not be sufficient to provide guidance towards adequate FBG conditions. In fact, we are highly concerned about the sensitivity of our predictions to disturbances and other uncertainties as will be shown. A simple, yet reliable model that accurately captures a decent surrogate of process performance and which can be analyzed thoroughly is of more value to us than a more complicated model predicting alternative properties with comparably low confidence.

In this paper we describe the results obtained applying a model that had been developed earlier (Lyngberg et al., 2016) for a project that involved a technology transfer of five legacy products from an older model granulator with discontinuous shaking of the air filters to a state-of-the-art granulator, while simultaneously scaling-up some of the processes. Due to the legacy nature of the products, a modern understanding of the design spaces (including univariate and multivariate knowledge about the process parameter ranges) was absent. Certain parts of the process had been included in previous regulatory filings such as the formulation and inlet air temperature ranges so based on agreements with the relevant health authorities, no changes were made to these parts of the process. The objective of the modeling part of this project was to explore the process parameters at pilot scale and then use that data to train the model and determine the commercial scale parameter targets and ranges.

## 2 Materials and methods

### 2.1 Products

The compositions of the five products involved in the project varies greatly, from drug loads as little as 0.5 wt% in the core tablet to drug loads greater than 80 wt%. Likewise, a diverse range of excipients is used in the formulations, including lactose, microcrystalline cellulose, different types of starch, and so forth. In this text, we have assigned generic labels A to E to the formulations. The spray solutions applied in the granulations, too, are diverse, with some products being granulated with various polyvinylpyrrolidone binders of different polymer chain lengths and others using starch solutions of different compositions. Even binder preparation methods, were varied, sometimes starting from pre-gelatinized starch and other times involving a process whereby the starch suspension was heated to form a paste-like spray. Analogously to the product naming, generic labels are used in this text to distinguish different processes where necessary. Maximum batch sizes of 350 kg were planned at commercial scale whereas most pilot scale experiments were executed at around 60 kg.

### 2.2 Granulators

Two models from Glatt GmbH were used in the parts of the project described here, a GPCG 30 with a 145 L bowl at pilot scale and a WSG Pro 200 with a 1000 L bowl at commercial scale. Spray rate was monitored by a mass flow meter at both scales and the peristaltic pump was operated in closed loop control with the mass flow meter at both scales enabling the specification of a particular spray rate in the equipment’s operating software. Inlet air flow and inlet air temperature were similarly monitored and controlled. Exhaust air temperature and the pressure drops across the product screen and air filters were all monitored at both scales. Inlet air humidity could be controlled at both pilot and commercial scale with the ability to both add and remove moisture with steam and a cooling coil respectively.

### 2.3 Loss-on-drying (LOD) testers

At pilot scale, the loss-on-drying tests were performed using a heated balance from Mettler Toledo, model HE73. At commercial scale, the equivalent tests were performed with a different model of the same vendor, namely model HB-43-S. At both scales, a sample was pulled to fill a container which was then tightly sealed. At both scales, the test was then performed as soon as possible using a method involving heating a 10 g portion of the sample to 105 °C for 15 min and determination of the moisture based on the difference between the initial and final weight.

## 3 Theory

### 3.1 Basic model

As stated earlier, fluid bed granulation is a complex process with a large number of mechanisms and factors that make this process difficult to model in its entirety. According to the general concept that 80% of the outcome can be achieved by 20% of the work (cf. “Pareto principle“), it should be favorable to apply a model that aims to describe a simplified set of surrogate variables, such as the LOD profile, if it can be shown to be a useful and scale-independent measure of the process performance within a sizeable region of the parameter space.

Still, it is clear that other factors, such as the droplet dispersion or the fluidization behavior and therefore equipment geometry, batch size, etc. are critical for a robust process. We believe that reconciling these contradictory facts can be achieved by bringing these other factors ‘under control’ to the extent that the main factor that remains is the LOD. This can be accomplished by using process expert know-how and statistical analysis of historical data and is facilitated by the fact that comparably few process parameters have a dominant effect. For example, in a given equipment fluidization is mainly controlled by the airflow rate, while spray cone width and droplet size are governed by the combination of spray rate and atomization pressure. The resulting considerations provide a set of constraints within which our simple model can operate.

#### 3.1.1 Main set of ordinary differential equations

**Fig. 1:**
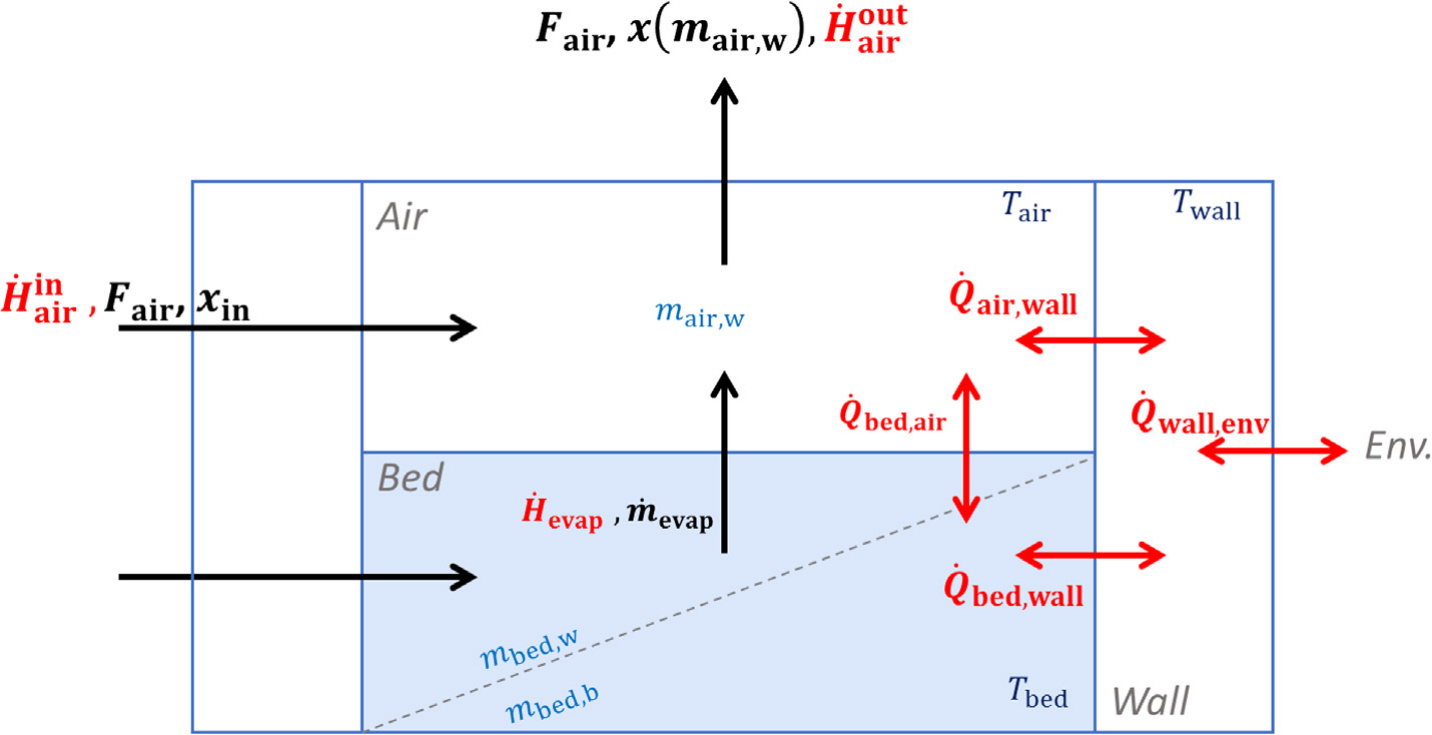
Schematic of the considered compartments and the mass and energy flows.

In the following, we briefly revisit the main principles and equations of the model, previously reported elsewhere (Lyngberg et al., 2016). The fluid bed system is described as a mass- and energy balance over two perfectly mixed compartments, as visualized in Fig. 1. The first compartment represents the ‘bed’ and contains all solids (incl. all API, excipients, binder, etc.) and the liquid water, while the second represents the humid air. Specifically, the ordinary differential equations (ODEs) governing the mass flow in and out these compartments are given by

(1) $$\begin{array}{c} \frac{dm_{bed,w}}{dt}={\left(1-w_{s}\right)F}_{spray}-{\dot{m}}_{evap} \end{array}$$

(2) $$\begin{array}{c} \frac{{dm}_{bed,b}}{dt}=w_{s}F_{spray} \end{array}$$

(3) $$\begin{array}{c} \frac{dm_{air,w}}{dt}=F_{air}\left(x_{in}-x\left(m_{air,w}\right)\right)+{\dot{m}}_{evap} \end{array}$$

where $m_{bed,w}$ and $m_{air,w}$ in Eqs. (1), (3) represent the mass of water in the bed (in liquid phase) and in the air (gaseous) respectively; $m_{bed,b}$ refers to the mass of solid binder, the only solid changing in mass over time. Furthermore, F denotes flowrates of air and spray, whereas $w_{s}$, $x_{in}$ and x are the solid weight fraction in the binder, the inlet- and the outlet air humidity, respectively. Finally, ${\dot{m}}_{evap}$ is the rate of evaporating water (see Section 3.1.2 for details).

Similarly, the equations describing the change in temperature for the bed, $T_{bed}$, is

(4) $$\begin{array}{c} \left(m_{bed,s}c_{p,s}+m_{bed,w}c_{p,w}^{l}+m_{bed,b}c_{p,b}\right)\frac{{dT}_{bed}}{dt}={\dot{H}}_{spray}-{\dot{H}}_{evap}-{\dot{Q}}_{bed,wall}-{\dot{Q}}_{bed,air}-\left(T_{bed}-T_{0}\right)\left({\dot{m}}_{bed,w}c_{p,w}^{l}+{\dot{m}}_{bed,b}c_{p,b}\right) \end{array}$$

Here, terms using $\dot{H}$ relate to changes in energy due to enthalpies of mass streams, while the $\dot{Q}$ terms represent heat transfer through conduction, convection, and radiation (see appendix for their definitions). The heat capacities $c_{p}$ are used in conjunction with the reference temperature $T_{0}$ in order to calculate the relevant enthalpies.

(5) $$\begin{array}{c} \left(m_{air}c_{p,a}+m_{air,w}c_{p,w}^{g}\right)\frac{dT_{air}}{dt}={\dot{H}}_{air}^{in}-{\dot{H}}_{air}^{out}+{\dot{H}}_{evap}+{\dot{Q}}_{bed,air}-{\dot{Q}}_{air,wall}-\left(T_{air}-T_{0}\right){\dot{m}}_{air,w}c_{p,w} \end{array}$$

(6) $$\begin{array}{c} m_{wall}c_{p,steel}\frac{dT_{wall}}{dt}={\dot{Q}}_{bed,wall}+{\dot{Q}}_{air,wall}-{\dot{Q}}_{wall,env} \end{array}$$

Lastly, we wish to highlight the fact that in practice, the inputs to the system from the perspective of the model, particularly, $F_{spray}$ and $F_{air}$, can deviate from their respective set points in the equipment. Reasons for this can be manifold and include, e.g., differences between the fluid properties during calibration and operation of mass flow meters (non-Newtonian vs. Newtonian), or the exact positioning of the sensor in the piping. This can lead to slight discrepancies between equipment trains and products that should otherwise behave identical. Our approach has been to introduce linear correction factors, α, such that:

(7) $$\begin{array}{c} F_{i}=\alpha_{i}F_{i}^{\mathrm{set}} \end{array}$$

for i∈{spray,air}. Note that, for the purpose of determining these factors, they can be treated as additional model parameters and therefore can be estimated via standard parameter estimation techniques. However, their interpretation is different. Empirically, we expect these correction factors to require updating infrequently, e.g., when moving from one site to another, and we expect those modifications to be small. To give an example, for the dozens of runs that have been modeled in this project, the correction factors are fixed for each product and never had to be modified.

#### 3.1.2 Equations for evaporation rate calculation

In order to solve Eqs. (1), (2), (3), (4), (5), (6), a number of heat- and mass transfer correlations, as well as state equations are required. In the following, we provide a brief overview of the most important equations related to the evaporation rate, ${\dot{m}}_{evap}$, the very core of our model and the location where the model parameters that need to be estimated from experiments have the greatest impact. Interested readers are referred to (Lyngberg et al., 2016) and the appendix for additional information.

For the water evaporation rate, it is found that the empirical correlation,

(8) $$\begin{array}{c} {\dot{m}}_{evap}=k\left(\theta_{e},\theta_{p}\right)A_{p}\left(\theta_{p}\right)\left(c_{w,sat}-c_{w}\right) \end{array}$$

yields excellent results in practice. In Eq. (8), $c_{w,sat}$ and $c_{w}$ are the concentration of water at the particle surface – assumed to be at equilibrium – and that in the chamber, respectively. The difference between the two constitutes the driving force behind the evaporation of water. Meanwhile, k and $A_{p}$ are the mass transfer coefficient and particle surface area, both functions of the two constant, non-negative parameters, $\theta_{i}$, in this model. Specifically, the area is given by

(9) $$\begin{array}{c} A_{p}=N_{p}\left(\theta_{p}\right)\pi \theta_{p}^{2} \end{array}$$

where $N_{p}$ is the number of particles in the system. Three important observations can be made about Eq. (9). First, parameter $\theta_{p}$ has a physical interpretation, namely that of ‘particle size’. Second, this size is assumed to be the same for all particles, i.e., the system is treated as monodisperse. Third, this size is treated as time-invariant. Clearly, neither the second nor the third assumption can actually be true in a granulation process and this work certainly does not claim otherwise. Rather, we point out that the resulting estimate of the available surface area is typically sufficient for the purpose at hand, as demonstrated in more detail in Section 4.1.2. It is a ‘wrong, but useful’ assumption.

The mass transfer coefficient is

(10) $$\begin{array}{c} k\left(\theta_{e},\theta_{p}\right)=k_{0}\left(\theta_{p}\right)\eta \left(\theta_{e}\right) \end{array}$$

where $k_{0}$ corresponds to the part of the transfer coefficient that depends solely on the Reynolds number (and therefore the particle size, $\theta_{p}$), and other terms not directly related to the moisture of the bed. The second factor in Eq. (10) defines an evaporation efficiency, given by

(11) $$\begin{array}{c} \eta =\left\{\begin{array}{cc} \exp\left(x_{\text{bed},\text{w}}\right)\theta_{\text{e}}^{-1} & \mathrm{if}\;\exp\left(x_{\text{bed},\text{w}}\right)\theta_{\text{e}}^{-1}⩽1\\ 1 & \mathrm{otherwise}\; \end{array}\right) \end{array}$$

with $x_{bed,w}=m_{bed,w}/\left(m_{bed,w}+m_{bed,s}+m_{bed,b}\right)$ as the loss on drying (in percent), implying that the mass transfer can be described as becoming effectively less efficient at lower moisture contents, a relationship which can be modified by adjusting the parameter $\theta_{e}$.

### 3.2 Output uncertainty analysis

In practice, the uncertainty in the measurable output of any physical system stems from a variety of sources, including measurement error, human error during execution of the process or the analysis, unforeseen changes or disturbances in the raw materials, the environment, or the equipment, and so forth (Vetter et al., 2015, Korakianiti et al., 2011). In the absence of these factors, which can be managed by proper definition of in- and output specifications, employee training, and (change) control procedures, the variability of operating conditions themselves is probably the dominant source of uncertainty. In fact, in the multi-purpose equipment commonly found in today’s pharmaceutical plants, low-level temperature, pressure, moisture, etc. controllers are rarely if ever tuned to any specific formulation in particular. Rather, the equipment is required to accommodate as large a range of process conditions as possible, which, together with the generally larger inertias due to the scale effects, can lead to a type of imperfect controller behavior that is rarely seen at lab-scale.

Similarly, the output of *models* of physical systems is affected by many of the same factors, including the input uncertainty outlined above. However, while the human element plays a smaller role, the precision with which the mathematical description and its parameters are actually determined becomes crucial. If model parameters cannot be estimated with sufficient precision, the resulting uncertainty in the prediction of the output can render the model useless in practice. This is true regardless of whether or not the model structure is intrinsically correct and unbiased.

In this work, our intent was to find a comparably simple, modular, and scalable approach to determine a worst-case overall output uncertainty for all products due to the two sources of error just described, input variability and parameter uncertainty. While there might be other, more involved ways to find maybe more exact measures of this overall uncertainty, we deem the trade-off between value and complexity of our approach as highly favorable.

#### 3.2.1 Input variability

In pharmaceutical industry a sound understanding of the sensitivity of the process output to changes in the process parameters is of utmost importance. In fact, all existing insight is typically recorded in a product-specific criticality report. The objective here is to obtain a deeper process understanding by complementing the know-how transcribed there with a large number of additional simulations, representing a variety of disturbance scenarios, specified as input profiles, that would be difficult to test experimentally (Korakianiti et al., 2011, International Conference on Harmonisation of Technical Requirements for Registration of Pharmaceuticals for Human Use, 2009). In order to generalize the applicability of our fluid bed granulation model as much as possible, all four inputs are considered to be of interest, that is, we investigate the effects of the inlet air temperature, the inlet air flow rate, the spray rate of liquid binder, and the inlet air humidity, resulting in a type of “risk fingerprint” for a given system that is indicative of its inherent robustness.

Of the four parameters mentioned, we deem the inlet air humidity to require special consideration. While it is typically measurable in the granulator, depending on the equipment setup for the entire plant, there may be limitations regarding its controllability. It is possible to have varying capabilities, ranging from no air humidity control at all, to condensers which can achieve a known minimum or maximum humidity, to full humidification and dehumidification where almost any physically feasible air humidity setpoint can be reached. As a consequence, the inlet air humidity may be a function of seasonal variation. To address this issue, the input variability was studied at three different inlet air humidity levels. The values were chosen based on climate data at the location of the receiving manufacturing plant, as well as the expected dehumidification performance by the installed condensers. On the driest days of the year, the absolute inlet air humidity, expressed as mixing ratio, was estimated to be as low as 1 g/kg. An upper limit of 10 g/kg was chosen based on the assumed capabilities of the condensers, while 5 g/kg was selected as ‘target’ humidity. The strategy for testing process robustness consisted of setting up simulations at each of the air humidity values, then performing various worst-case scenarios and assessing the impact on LOD maximum, that is the highest LOD value that is reached during the process – typically achieved at the end of the spray phase.

Note that our analysis, in which disturbed input profiles are fed into an existing model, is similar to others that have been performed in literature (Garcia Munoz et al., 2018), but it is not the only possible pathway. Alternatively, a study could be conducted via another modeling strategy, e.g., by accounting for the stochastic nature of inputs explicitly. While this would allow the variability of each process parameter to be described more realistically, it would require recasting of existing equations and a much more extensive analysis of historical data. It would inevitably depend on many external factors such as product formulation, FBG geometry, other operating conditions, etc. and ultimately render this analysis much less modular and increase complexity significantly. For all these reasons, it was chosen to rely on the simpler approximation outlined here instead, as it still constitutes a major improvement over the model-free approach, which is to not study these effects at all.

#### 3.2.2 Parameter uncertainty

In the following, it will be shown how the parameters have been estimated from experiments in a model calibration exercise. We then treat parameter uncertainty as has been done elsewhere, namely by using a Quasi-Monte Carlo technique to sample from the parameter space, followed by forward simulation and subsequent reconstruction of the uncertainty intervals (Vetter et al., 2015, Van Bockstal et al., 2017). Specifically, we use Sobol sets to perform sampling from multivariate Normal distributions characterized by the parameter estimate and covariance matrix, and test convergence by comparing changes in the resulting intervals over multiple iterations. Unphysical samples (negative parameters) are removed prior to running the simulations, and the minimum number of runs is 500. We further have used parallelization to speed up this process.

#### 3.2.3 Overall output uncertainty

**Fig. 2:**
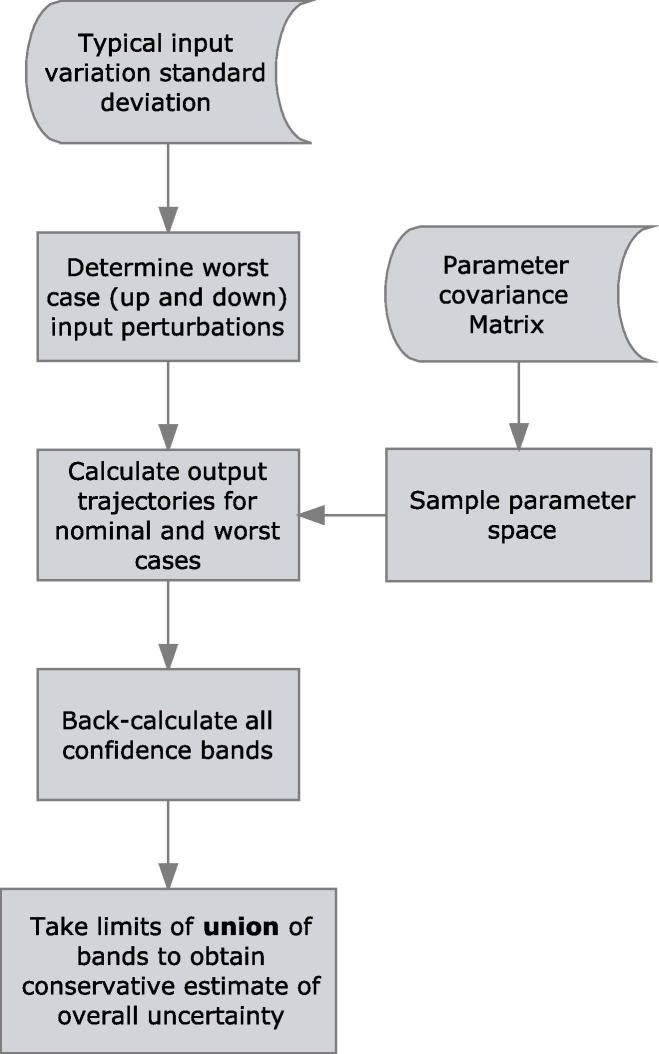
Schematic of total uncertainty determination workflow.

To obtain the overall uncertainty of the predictions, the two steps outlined in the previous sections are performed in sequence. First, the input variability assessment is performed and the set of inputs resulting in the most extreme profiles (judged by the maximum LOD) is selected. In the second step, the parameter uncertainty is computed for the two extreme runs as well as the nominal case and the overall confidence bound is estimated; a schematic overview of the procedure is provided in Fig. 2.

Note that this stepwise approach is not theoretically guaranteed to yield the most conservative band since no explicit proof is provided to demonstrate that there is no intermediate input set that yields a more extreme outcome. However, heuristically speaking the system is well-behaved and no instance of this occurring was ever observed. For this reason, and in order to save computational time, there is therefore no specific test against this, such as brute-force analysis of the parameter uncertainty for all input sets investigated.

### 3.3 Implementation

**Fig. 3:**
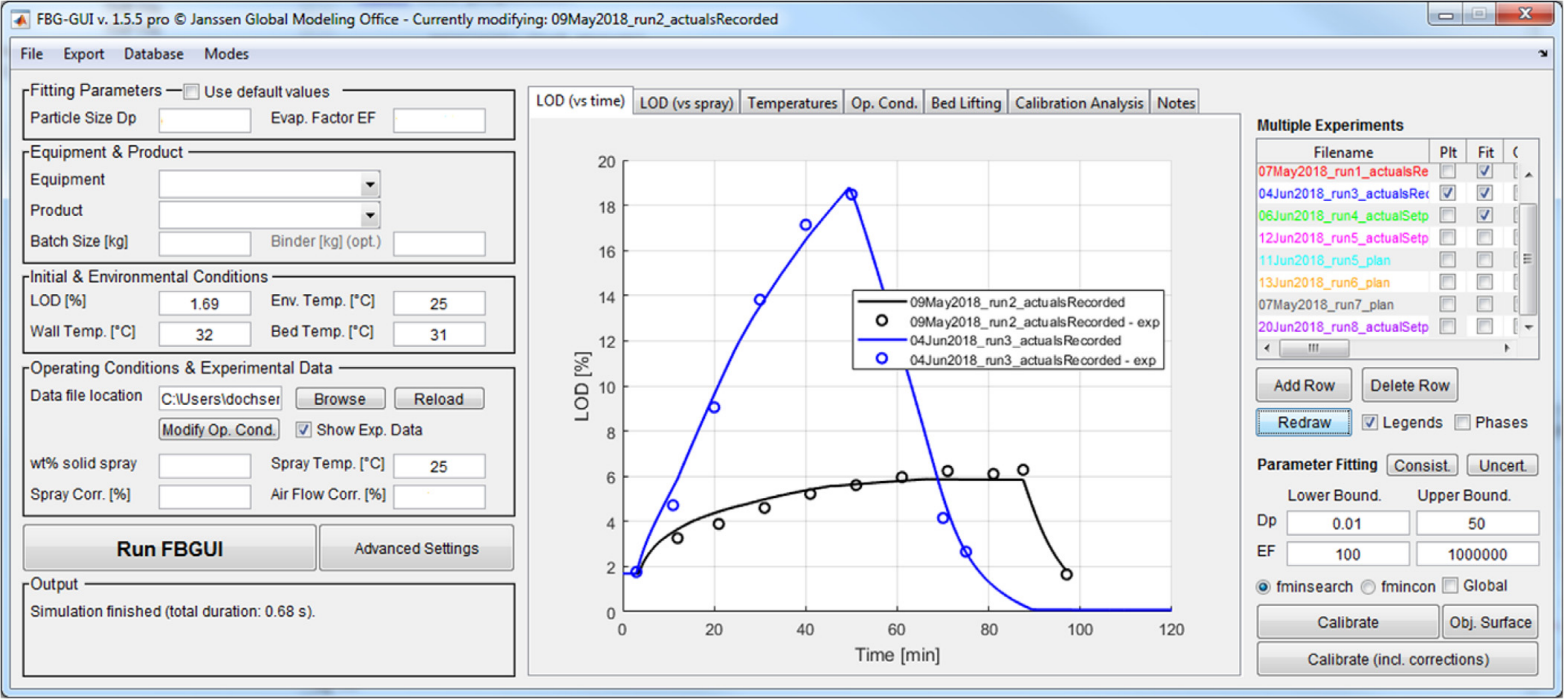
The graphical user interface.

The model is implemented as a class in Matlab and the main equations are solved using the stiff ordinary differential equation solver *ode15s*. The numerical solution of most problems requires at most a few hundred milliseconds on a standard-issue laptop, e.g, a quad-core i5-8350U running at 1.70 GHz and 16 GB of local memory (1 SODIMM × 16 GB @2400 MHz). Process parameters can be read out from tables, sets of simulations can be stored and exchanged via .mat files, and results can be exported and shared in the form of figures and tables. To facilitate interaction with the tool and allow for wider deployment also to non-modelers, a graphical user interface was developed, that allows to visualize, compare, and modify simulations on the fly (see Fig. 3).

## 4 Results

### 4.1 Model building

While the LOD model enables users to predict granule wetness quantitatively, in practice, not the entire moisture space leads to acceptable downstream in-process control properties and critical quality attributes. Simple binary correlation analyses correlating, e.g. maximum LOD and these in-process control properties or quality attributes suggest a significant correlation of the LOD profile with tablet hardness and time-dependent release data of dissolution.

This LOD design space may be inferred from historic data, e.g., from process characterization or validation reports. However, the covered ranges may be limited. Fortunately, since the model enables pilot-scale results to be scaled up to full scale, the effects of the moisture profile can be studied at pilot scale by taking the granules obtained from these batches into the down-stream operations and subjecting the final product to the standard quality testing. Note that two goals can be realized at once in pilot scale: exploring the LOD design space while simultaneously calibrating and validating the model.

#### 4.1.1 LOD design space

**Fig. 4:**
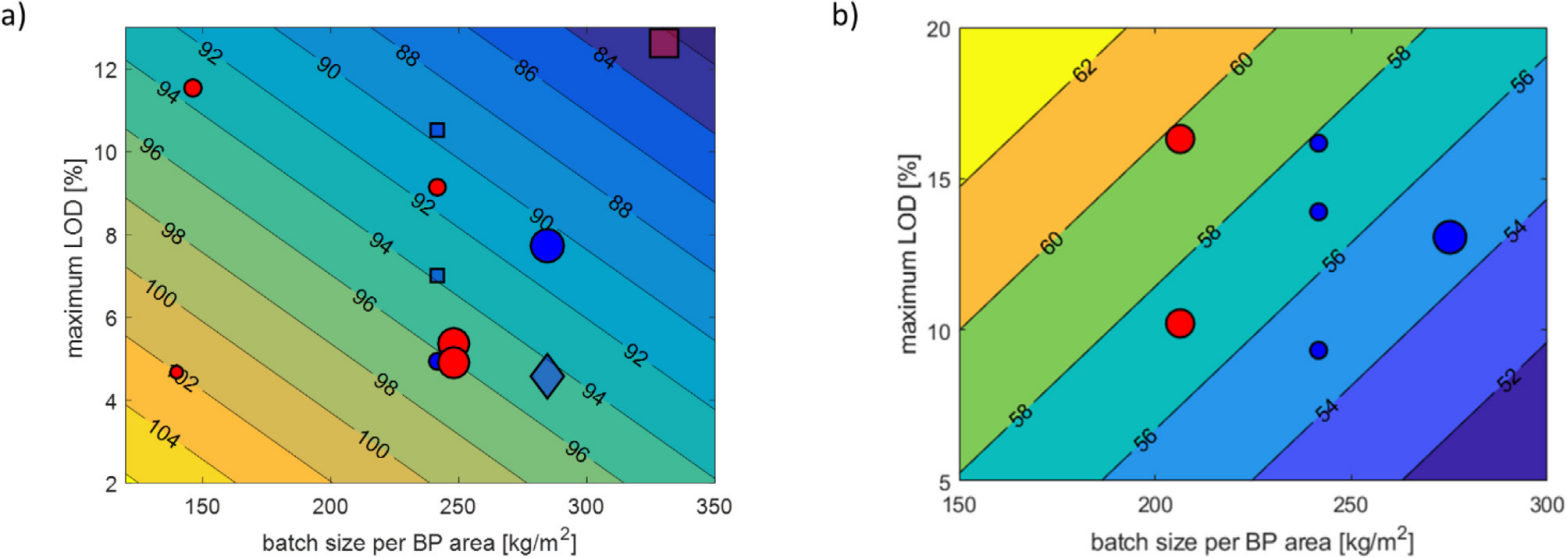
Influences of maximum LOD and batch size per bottom plate (BP) area on hardness (in N) for Products B (a) and E (b). Blue circles: batches manufactured using binder preparation method L; red circles: batches manufactured using binder preparation method X: grey squares: batches with hardness beyond specifications. The area of a circle or square is proportional to the batch size. (For interpretation of the references to colour in this figure legend, the reader is referred to the web version of this article.)

A higher maximum LOD (or a wetter batch) may result in softer or harder core tablets after compression (see Fig. 4), depending on the product of interest. The trends were summarized over multiple sites and from different batch sizes and equipment. Wetter conditions may favor the formation of a tighter and firmer binding of particles into granules for Product E, as a negative correlation between maximum LOD and tablet hardness is found for the same product in Fig. 4. In contrast, Product B exhibits opposite hardness-granule wetness dependence, in which the starch binding solution, which gels easily, plays a considerable role. The effect of LOD history on API release data for the final tablet products is more complicated – both clear trends and large error-to-signal cases have been observed. These analyses suggest that in the general case the maximum LOD or the wetness of a batch must be controlled within a range to ensure that properties such as hardness and dissolution pass the quality specifications.

#### 4.1.2 Model calibration and validation

Model training and testing follows the general guidelines and perspectives outlined by regulatory bodies (FDA, 2012, Chaterjee et al., 2017) as well as internal procedures. Specifically, for this project, a minimum of three pilot-scale batches were carried out for each product to cover wet, target and dry granulation conditions. If all final tablet products pass the quality criteria, the boundaries of the design space were then defined by the largest and the smallest maximum LOD’s of these batches. To determine the parameters three pilot-scale batches were arbitrarily split into training set, consisting of two batches A and B, and a prediction set, consisting of batch C. In a first step, the LOD model is calibrated on batch A and the resulting parameters are used to predict the LOD trajectory of batch B. Second, the process is reversed, training the model on batch B and predicting batch A. These preliminary steps serve to gauge the overall consistency of the two sets of data. Finally, a third exercise is conducted in which the model is calibrated on the entire training set, that is, batches A and B, and this model is then used to predict the independent batch C, completing the validation. Throughout, all the training and prediction errors are computed in the form of the root mean square of the error (RMSE), e, a function of the difference between each measured sample i of the loss on drying, *x*_bed,w,_*_i_*, and its corresponding model prediction (here indicated by a hat symbol).

(12) $$\begin{array}{c} e=\sqrt{\frac{\sum_{i=1}^{n}{\left({\hat{x}}_{bed,w,i}-x_{bed,w,i}\right)}^{2}}{n}} \end{array}$$

In order for a calibration/validation procedure to be considered successful, i.e., for the model to be labeled an acceptable virtual representation of the physical system, these errors are compared to previously defined acceptance criteria. The exact values for these thresholds are different for training and prediction sets and stem from an analysis performed during the original development of the tool. There, the LOD model had been used to train and predict more than twenty-five batches of various products, ranging from 5 to 250 kg to test robustness. The acceptance criterion for the calibration was established as the average calibration RMSE plus twice the standard deviation of all the calibration exercises over the historic batches; similarly, the acceptance criterion for validation was defined as the average prediction RMSE plus twice the standard deviation of the historic testing exercises (final threshold for calibration: 0.279; for validation and verification: 0.664).

**Fig. 5:**
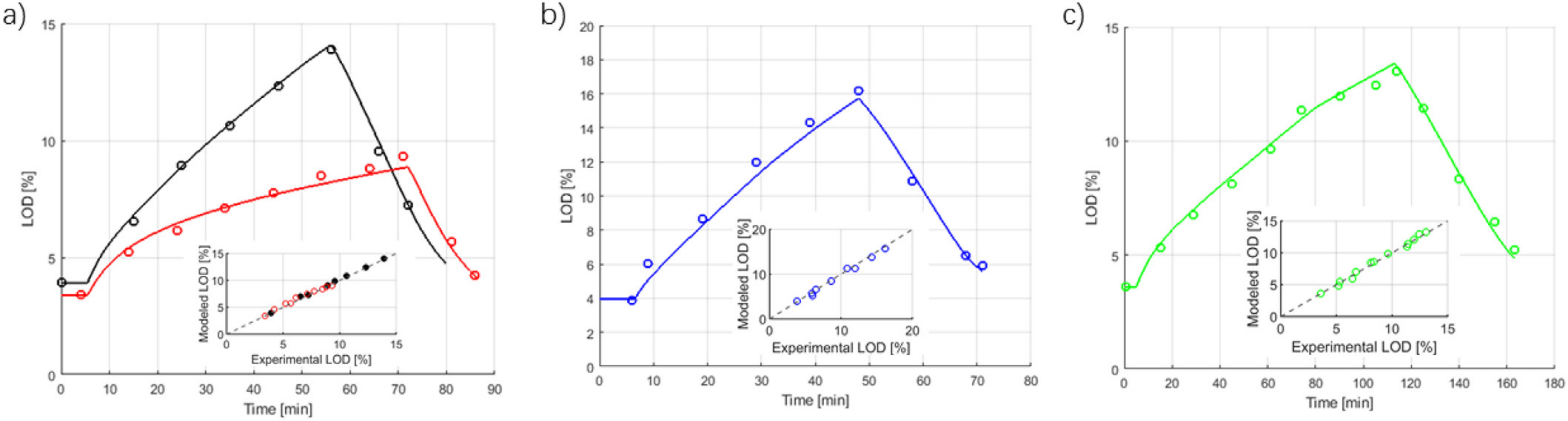
(a) Overall calibration over two pilot-scale batches (58 kg each, RMSE = 23.5%). (b) Validation on one pilot-scale batch (58 kg, RMSE = 53.9%). (c) Validation of one commercial-scale batch (216 kg, RMSE = 29.0%). Product E is used from Panel (a)–(c). Inlets represent the parity plots for each case.

Plots illustrating the agreement between model calculations and the sample measurements are given for one case, product E, in Fig. 5. The initial flat, rising and declining parts of the curve represent premixing, spraying and drying phases, respectively. Spraying and drying phases can be further divided into sub-phases to allow more gradual changes in the process conditions. For the batches in Fig. 5, the larger contributions to the RMSEs come from the errors of the points with higher LODs. Observed in several other batches (not shown here), RMSEs can also be accumulated significantly from the drying phase, in which the dynamics become faster and consequently are harder to capture both because a higher sampling frequency might be needed and because small errors in the recorded sampling time can have a significant impact on the overall error.

**Fig. 6:**
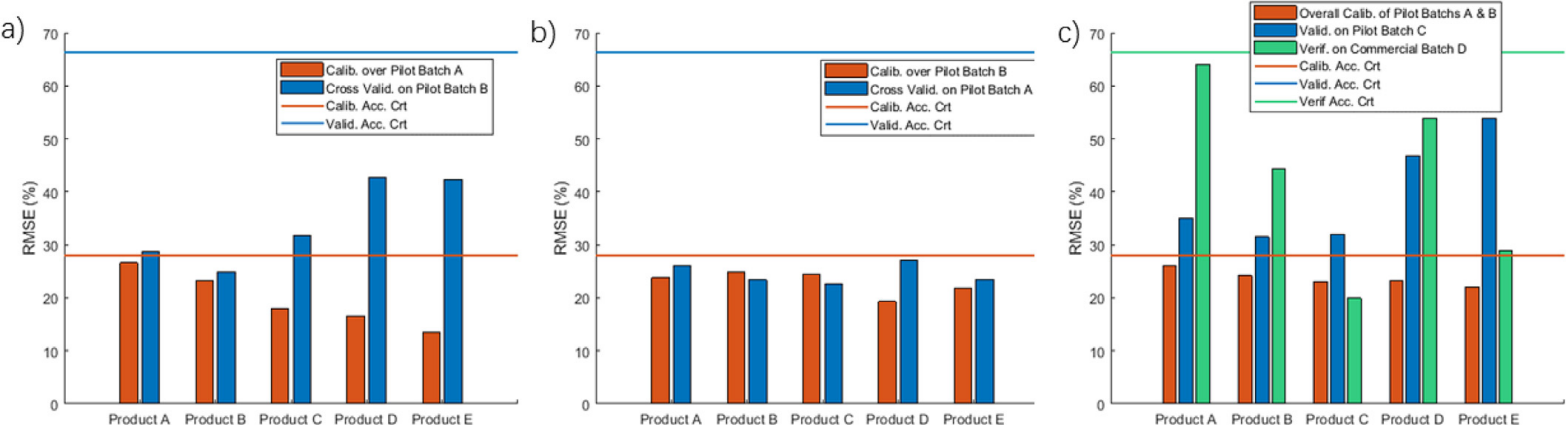
Errors for the three calibration and validation exercises at pilot scale, and one prediction at commercial scale, as well as the acceptance criteria for calibration and validation. (a) exercise in which the model is trained on data of batch A and cross-validated with data of batch B; (b) exercise in which the model is trained on data of batch B and cross-validated with data of batch A; (c) exercise in which the model is trained on data of batches A and B and validated and verified with independent batches C and D. Note that all quantities are given in percent of LOD%, i.e., all encountered errors average well below 1 LOD%. The acceptance criteria are 0.279 for calibration and 0.664 for validation and verification.

Upon successful calibration and validation of pilot-scale batches, the model was used to design the process parameters, namely, inlet air temperature, inlet air humidity, airflow rate and spray rate at commercial scale. To verify the model’s ability to predict LOD over granulators and scales, the resulting prediction was compared with experimental data of a fourth batch D, ensuring that also in this case the RMSE is below the validation threshold. The RMSE’s of all the calibration and validation exercises for all products in our technical-transfer project are presented in Fig. 6 together with the associated acceptance criteria. Given that all errors lie well below their acceptance limits, it suggests a good quantitative predictability of the moisture by the model in the granulation design and execution, with a prediction error significantly smaller than 1 LOD% on average.

While the model enabled a controllable granulation, the granules of the pilot- and commercial scale batches still need to go through all downstream unit operations, with each operation parameters designed by relevant models or tools. The final products all passed the quality specifications, suggesting the granulation moisture range covered in the calibration and validation exercise should be used as a confirmed design space in future design or improvement.

### 4.2 Input variability study

Once the model for a particular product has been calibrated and validated, it can be used to explore various process scenarios in silico. In the following, an overview of the scenarios that have been tested during the input design space analysis will be provided. Subsequently, the added value shall be demonstrated by providing results for two representative products. The first represents a robust process that can tolerate comparably large disturbances. The second process is more sensitive, with a maximum LOD that is strongly impacted by changes in the granulation operating conditions.

#### 4.2.1 Scenarios considered

**Fig. 7:**
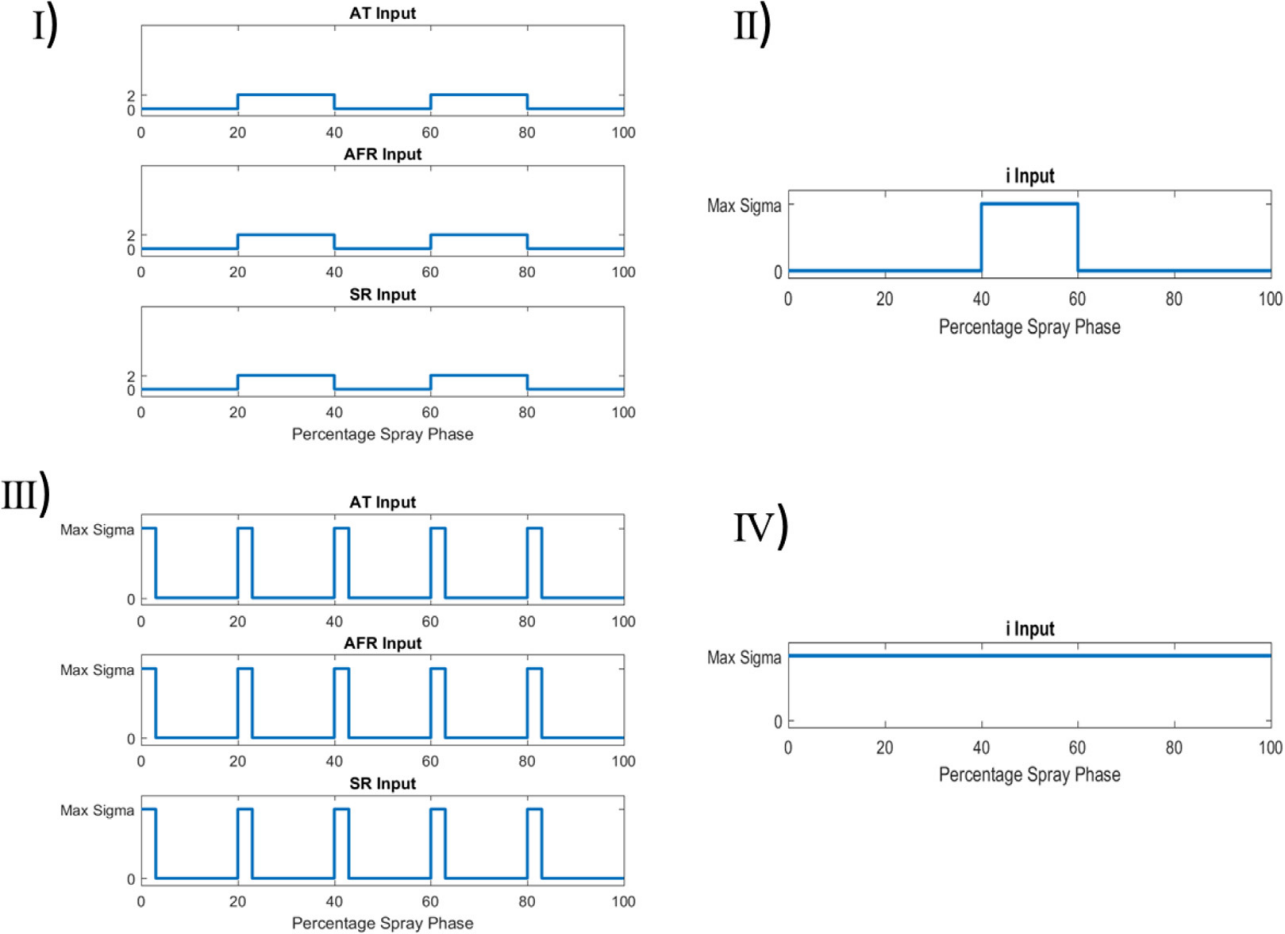
Input parameter deviations for the scenario sets. Vertical axis represents size of deviation in units of standard deviations for that input parameter. Scenario sets I and III vary the number of parameters deviating in one simulation, whereas scenario sets II and IV vary one parameter per simulation. Scenario set I has a fixed deviation size (2 sigma) whereas the other scenarios search for the maximum allowable deviation size for each simulation. Scenario sets III and IV have fixed deviation patterns, whereas sets I and II go through all k-combinations of parameter and number of phases deviated. AT: inlet air temperature; AFR: inlet air flow rate; SR: spray rate.

In general, this study aims at exploring four dimensions which determine the type of input deviation: the magnitude, the timing, the (total) duration of the disturbance, and the number of process parameters simultaneously affected during a disturbance. More specifically, scenario set I explores the effects of small magnitude deviations for a large range of deviation durations and parameter combinations. It is a reasonable proxy for a large variety of minor issues that may plague a set of equipment and a good first indicator for potential issues. Scenario set II aims to isolate the time of deviation phase and the parameter deviating associated with maximum LOD excursions. This is a useful scenario to determine the time-sensitivity of the maximum LOD on disturbances. Scenario set III explores the effect of very short, but potentially large magnitude disturbances, e.g., caused by a badly tuned low-level controller. Lastly, scenario set IV was meant to simulate a calibration error. The air temperature probe, air flow meter, or the mass flow meter at the liquid binder pump are all sensors that must be calibrated to a certain accuracy and frequency. This scenario set explores how sensitive the process is to certain parameters and gives an indication concerning how accurately the sensors must be calibrated. Table 1 contains an overview of all scenarios studied during the input design space analysis, carefully selected to represent a variety of situations, while Fig. 7 provides example visualizations of the disturbance profiles for each scenario set

**Table 1:** Overview of Input Variability Study Scenarios. Factors that are varied within a scenario are typed bold.

	Disturbance Magnitude	Disturbance Timing	Single Disturbance Duration	Overall Disturbance Duration	# of Process Parameters Affected	Interpretation
Scenario Set I	2σ	*N/A*	20%of spray	20,40,60,80,100%**of spray**	**1**–**3**	Study effect of minor equipment malfunction
Scenario Set II	(1-30)σ	**Phase 1**–**5**	20%of spray	20%of spray	1	Study whether effects of disturbances are time-variant
Scenario Set III	(1-20)σ	N/A	5min	30min of spray	**1**–**3**	Study effect of short, multi-variate disturbances
Scenario Set IV	(1-20)σ	N/A	100%of spray	100%of spray	1	Study effect of equipment calibration off-set

As stated earlier, all simulations are performed at three different humidity levels. If out of the remaining three process parameters more than one is varied (see second to last column in Table 1), all k-combinations are analyzed separately. Disturbances are always distributed uniformly over the entire spray phase and their magnitudes measured in terms of typical standard deviations (a unique value for each process parameter). To reduce the number of simulations somewhat and because this is a worst-case assessment, it is assumed that all deviations in a given run have the same qualitative impact, either purely acting to increase or decrease the maximum LOD. In the following, we refer to these disturbances as “wet deviations” and “dry deviations”, respectively. Clearly, random disturbances are more likely to cancel each other out. By ensuring that the final processes are robust even to these highly biased, improbable deviations, our confidence that the final process is capable of maintaining the LOD within the design space under *real* disturbances is high.

Lastly, we further rank scenarios based on their perceived likelihood by giving each executed scenario a deviation score, which will be relevant for scenario sets I and III in particular. Here, this number is defined as the product of the number of parameters varying and the number of granulation phases that are affected by the deviation. The resulting number is related to the overall estimated frequency of occurrence, useful, e.g., during failure mode effects analysis (FMEA) (Chaterjee et al., 2017). Here, higher scores reflect *lower* expected frequency, but also higher probability that the process might move outside the LOD design space. Points where simulations start to go outside LOD design space are of particular interest: if this occurs already at low scores, it indicates a lack of process robustness with regards to input variability.

#### 4.2.2 Performance metrics

In order to assess the robustness of a given process or product, two types of metrics are being used in the following. First, for scenario set I in which the disturbance magnitude is not varied, the fraction of simulations that resulted in out-of-LOD-range moisture profiles is computed and analyzed as a function of the deviation score. Low fractions reached at high scores are indicative of a robust process, as this implies that only a small number of unlikely, exceptional events – that can be investigated further – might realistically lead to LOD deviations in either direction, provided the magnitude of the disturbance itself is bounded.

The second metric is used for scenario sets II to IV, for which the disturbance magnitude is varied. In these cases, the *maximum tolerable input deviation* expressed in process parameter standard deviations, σ, is searched for in a given range. A robust process should be able to tolerate significant deviations before leaving the LOD design space. In the case of univariate disturbances, i.e., scenario sets II and IV, we further break these results down to the level of the individual process parameter. In scenario set III, where disturbances are multivariate in the input space, we group simulations based on their deviation score and provide error bars instead.

#### 4.2.3 Case study I: robust process (product E)

**Fig. 8:**
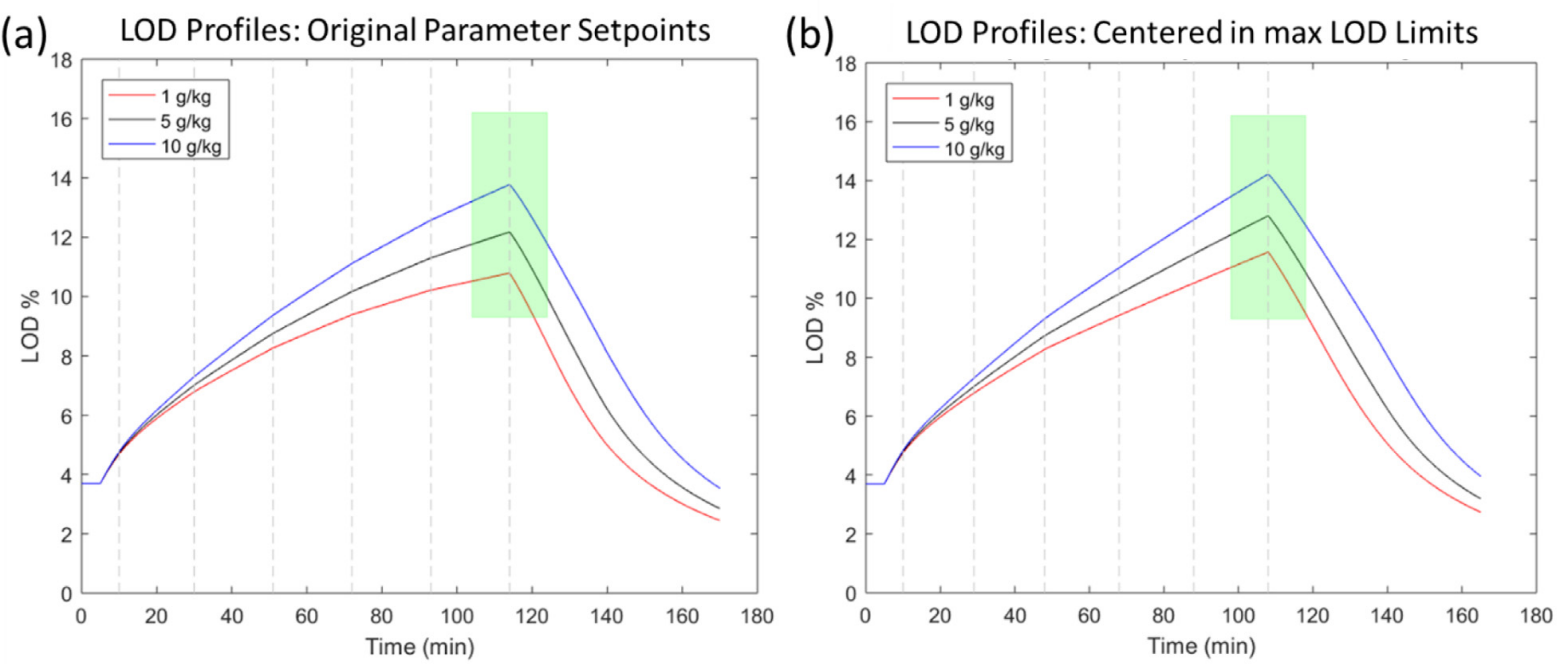
(a) Maximum LOD profiles for three air humidity levels for original operating parameter setpoints. (b) Maximum LOD profiles after midrange air humidity is centered in maximum LOD range. The green area represents the maximum LOD design space.

Process analysis for product E began by tracking maximum LOD over the course of the simulated process for the initially proposed target operating conditions. It became immediately clear that the maximum LOD is not well-centered within the range, reducing overall capability of the process. Therefore, the spray rate was modified, while keeping total binder sprayed constant, to adjust the maximum LOD of the midrange humidity level in the center of the maximum LOD range was the first adjustment. The LOD profiles before and after this change for the nominal and the two extreme humidity levels are presented in Fig. 8.

**Fig. 9:**
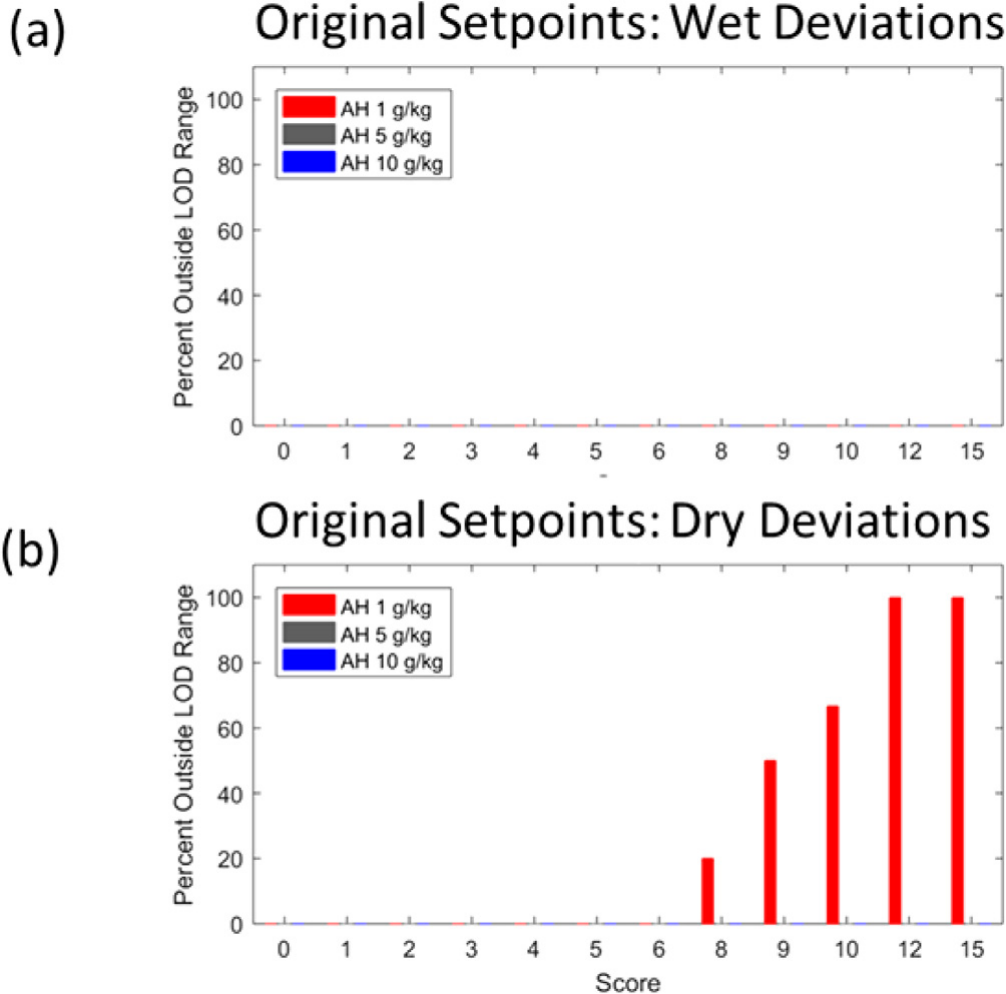
Results for scenario set I for product E. (a) Results for wet deviations on initially proposed target. No simulations of any score were outside the LOD range, therefore all bars have value 0 and are not visible. (b) Results for dry deviations on the initially proposed target. Performing the same deviations for the centered process resulted in no simulations being outside the LOD range. For each score, the percentage outside LOD range was 0%, therefore those results are not shown, but look like (a).

To see the impact of this slight shift, the results obtained during testing of scenario set I can be analyzed, as is done in Fig. 9. Shifting the midrange humidity LOD profile to the center of the LOD range has the benefit that, even the worst-case scenario with a score of 15, in which air flow rate, inlet air temperature, and spray rate deviated by two standard deviations from the target for the entire length of the spray phase, did not cause the maximum LOD to depart from the acceptable range.

**Fig. 10:**
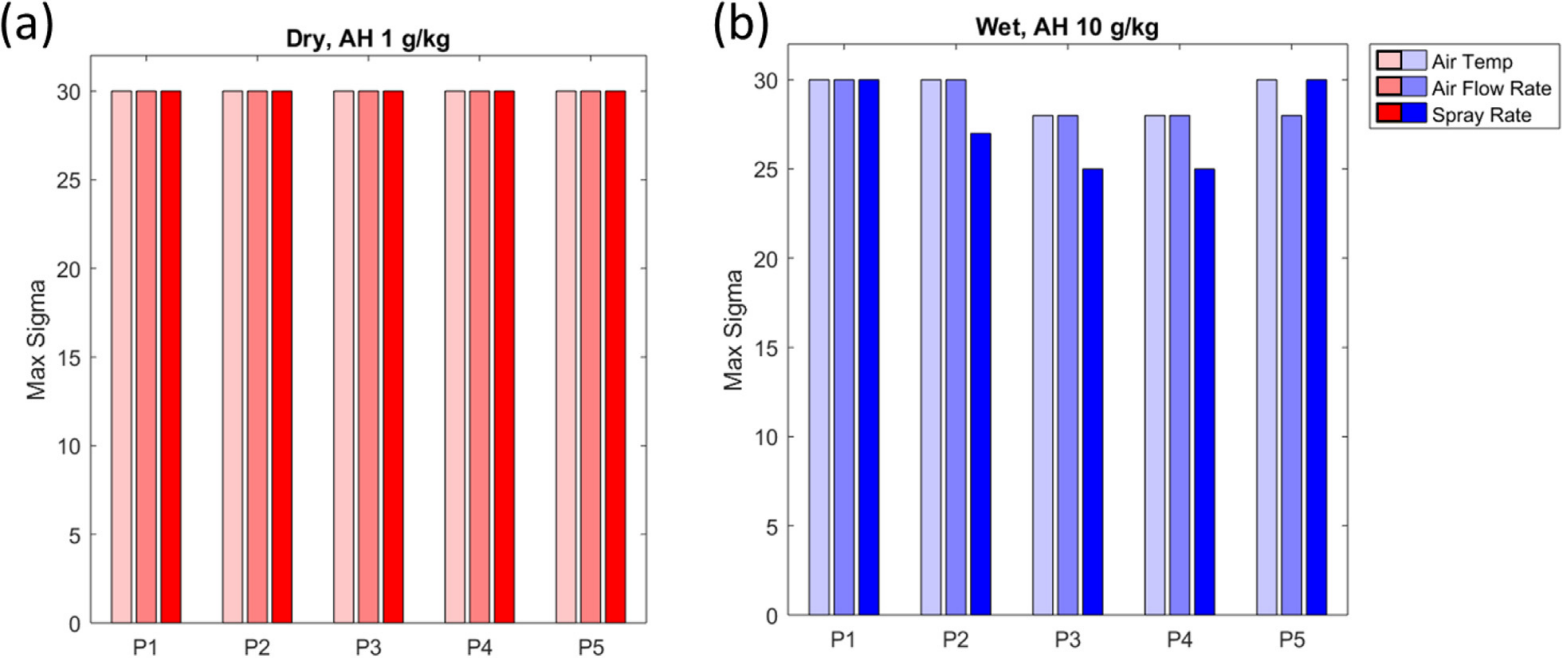
Results for scenario set II for product E. (a) Results for simulations with 1 g/kg air humidity pushed drier. (b) Results for simulations with 10 g/kg air humidity pushed wetter. Wet deviations in spray rate in the middle of the process have largest impact on maximum LOD. P1, P2, P3, P4, and P5 are the 1st, 2nd, … 5th subphase of spray, respectively.

Exploration of the impact of which parameter, when, and how large the deviation was which would cause the maximum LOD to go beyond the desired bounds is also of interest and is analyzed in scenario set II; the results are shown in Fig. 10.

It is evident that wet deviations during the middle phases of the process required a smaller magnitude of deviation to push granulated product out of the maximum LOD design space. In addition, this product appears more robust to dry deviations than wet deviations, although the required deviations to push the process out are very large in either case.

**Fig. 11:**
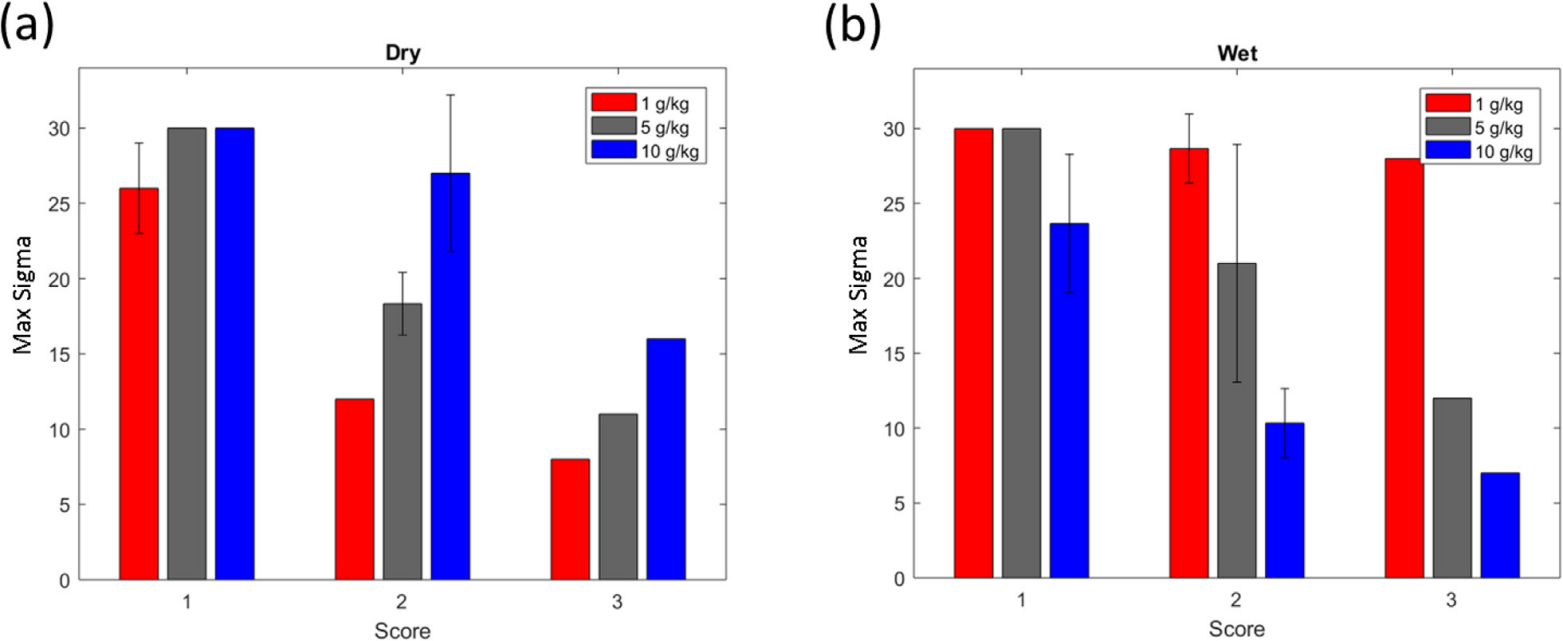
Results for scenario set III for product E. In these scenarios, score indicates how many parameters were deviated. (a) Results for simulations with dry deviations. (b) Results for simulations with wet deviations. In the case where all simulations reached 30 standard deviations without being outside the maximum LOD range, the standard deviation is zero and the associated error bar is missing. Bars associated with score 3 do not have error bars as they contain only one simulation each.

The next analysis, scenario set III, investigates the effect of six deviations during the spray phase, assuming the duration of each is five minutes; results are shown in Fig. 11. With only one parameter being deviated (score 1), the process requires significant deviations (greater than15 standard deviations) to be pushed outside the design space. And even when three parameters deviate simultaneously (score 3), there is around eight standard deviations of buffer from the target setpoints before the maximum LOD departs from the acceptable range.

**Fig. 12:**
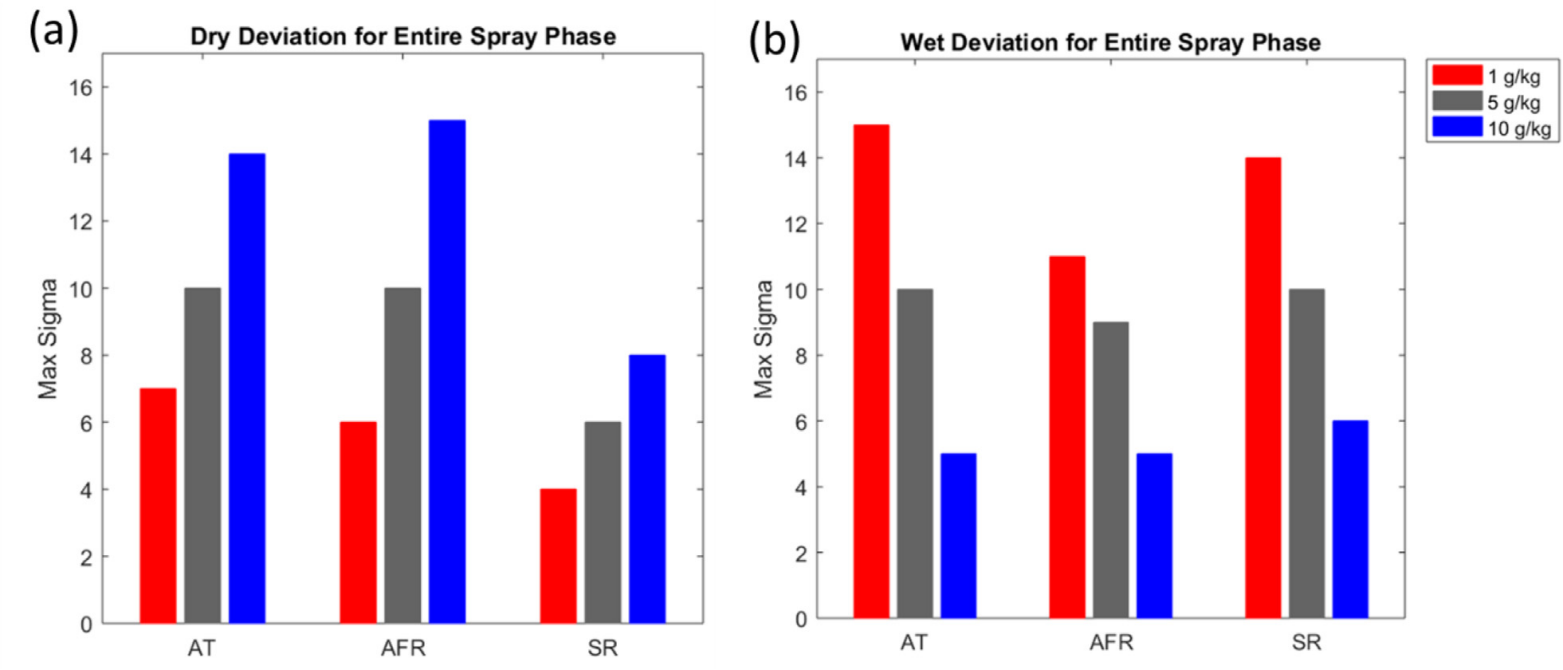
Results for scenario set IV for product E. (a) Results for simulations with dry deviations. (b) Results for simulations wet deviations. AT: inlet air temperature; AFR: inlet air flow rate; SR: spray rate.

The final scenario set IV was performed to see how large a deviation could be for the entire length of the spray phase. As seen in Fig. 12, the process is reasonably robust towards both wet and dry deviations for all the adjusted parameters. Whereas the process is most vulnerable in the case of a spray rate off-set during the dry season (third red column from the left in Fig. 12.a)), even then the necessary deviation is a highly noticeable four standard deviations.

In summary, this process exhibits significant robustness towards all disturbances that were investigated, regardless of environmental conditions. This implies a low risk of encountering issues with the process itself both during the transfer of the process as well as during commercial production.

#### 4.2.4 Case study II: sensitive process (product B)

**Fig. 13:**
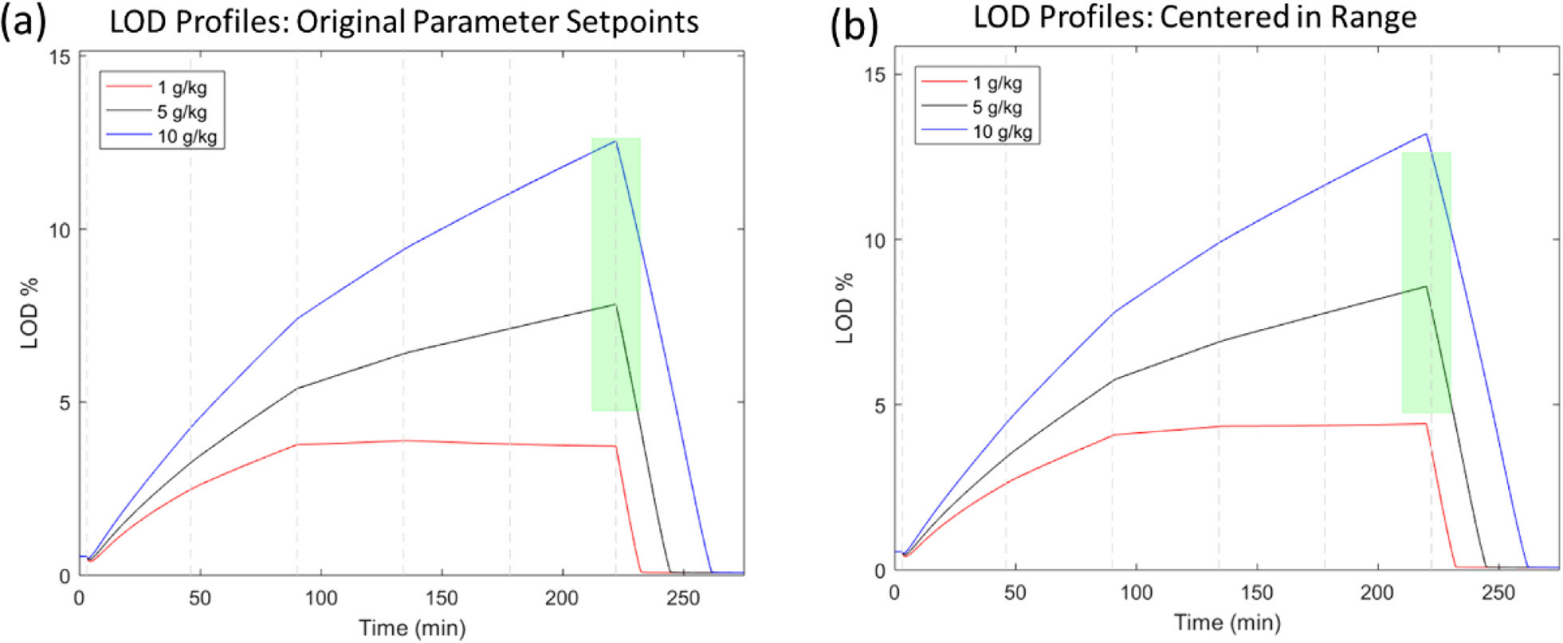
(a) Maximum LOD profiles for three air humidity levels. (b) Maximum LOD profiles after mid-range air humidity is centered in maximum LOD range. Adjustment made by increasing spray rate slightly for the entire spray phase. The green area represents the maximum LOD design space.

A similar analysis strategy to the above was used for product B. In a first step, the target process had to be centered in the maximum LOD range, the width of which is comparable to that of product E. The results of the initially proposed and the centered process are presented in Fig. 13. As can be seen, in contrast to the previous case, the varying air humidity has a significant impact on the maximum LOD. In fact, by centering the maximum LOD of the midrange humidity level, the maximum LOD at the high and low bounds of air humidity are outside of the acceptable range. This implies a risk for the manufacturing of this product under extreme weather conditions even without any disturbances.

**Fig. 14:**
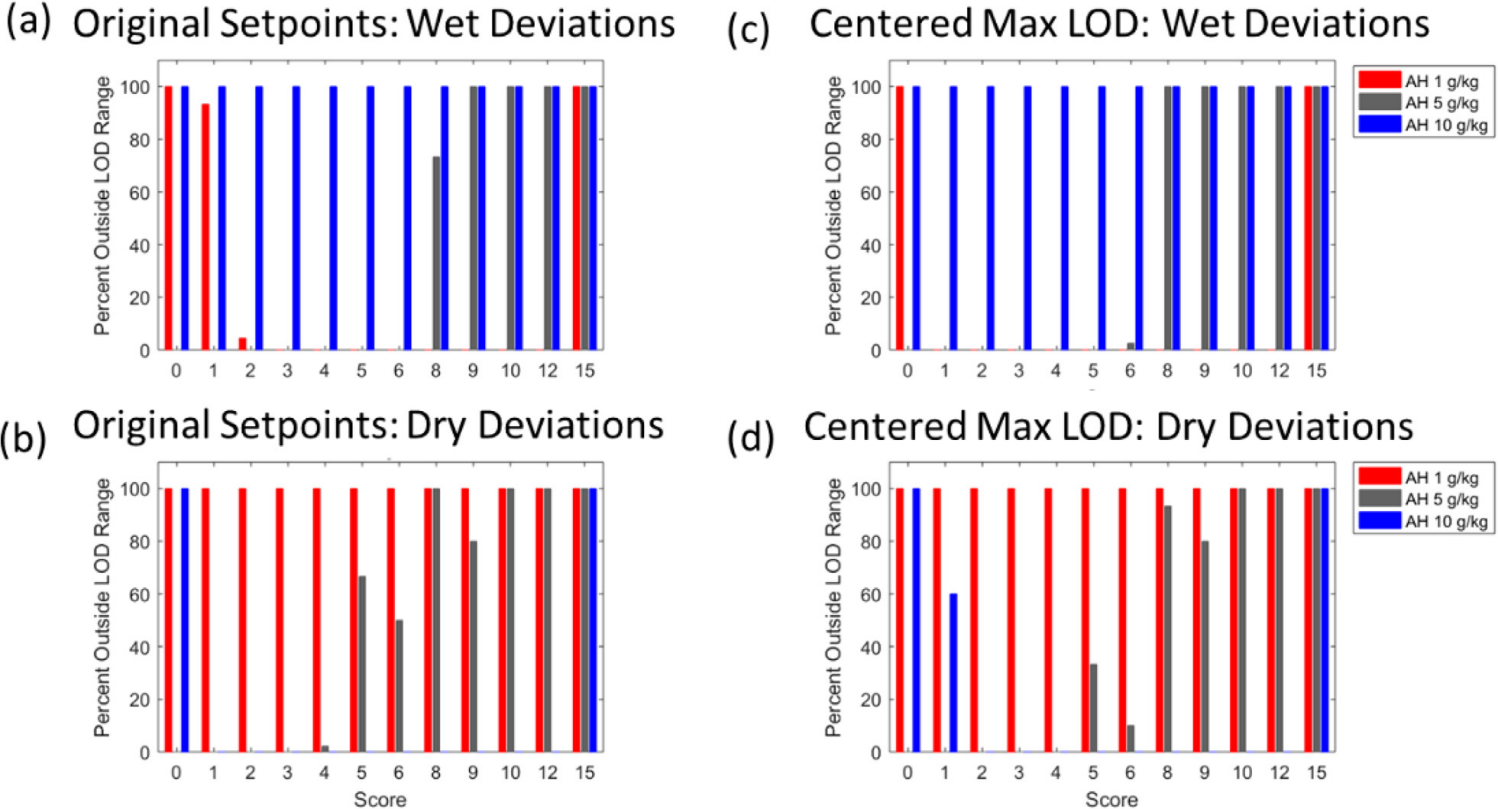
Results for scenario set I for product B. (a) Results for wet deviations on initially proposed target. (b) Results for dry deviations on initially proposed target. (c) Results for wet deviations on centered process. (d) Results for dry deviations on centered process.

Still, the harmful consequences of any additional process deviations are investigated first through scenario set I. As shown in Fig. 14, the process is not robust to changes in air humidity and the other deviated operating parameters. As is to be expected, *all* simulations at the upper and lower bounds of the air humidity range do not meet maximum LOD requirements, even the ones in which no parameters are deviated (score equal zero). Under mid-range air humidity conditions, the process shows to be capable of handling deviations up to score 5, which represents one parameter deviated by two standard deviations for the entire spray phase. We can also observe in Fig. 14.a) how the initially too dry process taking place at dry inlet air humidities is initially pushed *into* the LOD acceptable range by wet deviations before it is finally leaving it again at the extreme conditions represented by score 15. Clearly, the resulting robustness is vastly inferior to that of product E.

**Fig. 15:**
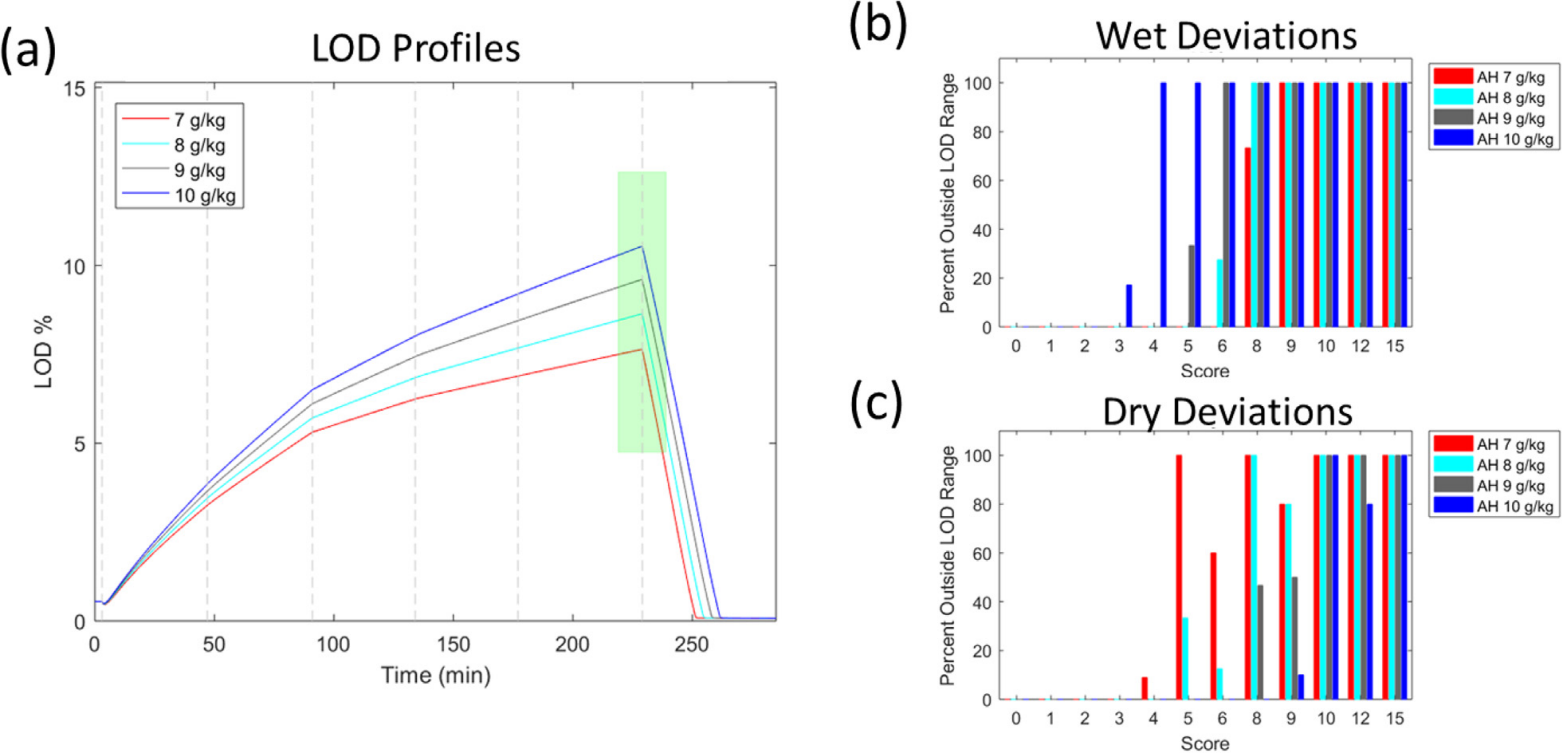
Results for scenario set I for product B using a humidity-dependent recipe. This analysis is done over the range of 7 g/kg to 10 g/kg. In this case, score represents the number of parameters impacted and the number of phases which are deviating. (a) Maximum LOD profiles for air humidity levels. (b) Results for wet deviations. (c) Results for dry deviations.

The strong sensitivity of this product to fluctuations in the incoming air humidity constitutes a major challenge as it implies high risk of maximum LOD excursions either during transfer or commercial production. Options for change control procedures were constrained due to specifications in the original filing of the process and the legacy nature of the product. However, one proposed solution satisfying the existing constraints is to implement feed-forward control of the process based on the environmental conditions (Garcia Munoz et al., 2010). To this end, the air humidity range was split into three separate groups: (1–3 g/kg), (4–6 g/kg), and (7–10 g/kg). By creating humidity-dependent recipes, we were able to adjust the spray rate and center the maximum LOD in the design space for each of the three groups. For the sake of brevity, only the results accounting for air humidity levels between 7 and 10 g/kg are presented. The LOD profile and the result of the small magnitude and long duration deviations, as prescribed by scenario set I, are presented in Fig. 15. The ability to handle small magnitude deviations up to a deviation score of three is recovered, although the process appears prone to maximum LOD excursions due to wet deviations. While this is not as robust as the previous example, it is a significant improvement over the original process. By study of Fig. 15.c), we can further notice that the behavior of the robustness metric is not necessarily monotonic with the increase in the score value. This turns out to be an early warning sign that the timing of the disturbance may matter more strongly than for the more robust product (cf. scenario set II).

**Fig. 16:**
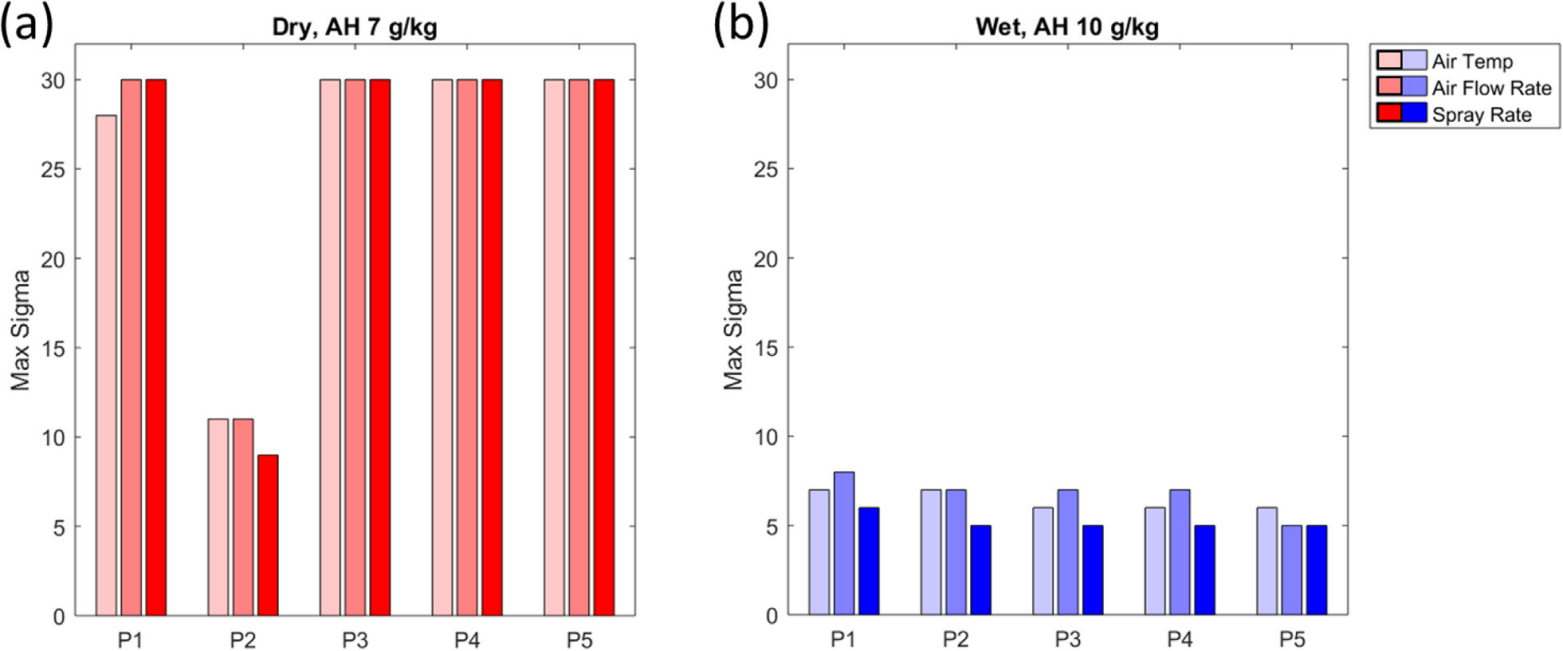
Results of scenario set II for product B using a humidity-dependent recipe. (a) Dry deviations for air humidity (AH) of 7 g/kg. Phase 2 deviations have greatest effect on max LOD because deviations in this phase prevent LOD of product from reaching max LOD. (b) Wet deviations for AH 10 g/kg.

Analysis of the results for scenario set II for this adapted process, provided in Fig. 16, are in agreement with previous results in that they indicate that wet deviations are more likely to push granulated product out of the LOD design space on average. Interestingly, for dry deviations of any parameter, phase 2 appears to be a critical time. This is due to the process reaching its maximum LOD in that phase in the case of a dry deviation, a consequence of the specific recipe used. Deviations in the previous phase allow enough time for the process to recover, while deviations in subsequent three phases cannot affect the maximum LOD any longer. Scenario set II has thus alerted us to the fact that wet deviations pose a greater risk overall, but that the effect dry deviations is not time-invariant. Ideally, this ought to be addressed by modifying the process parameter setpoints.

**Fig. 17:**
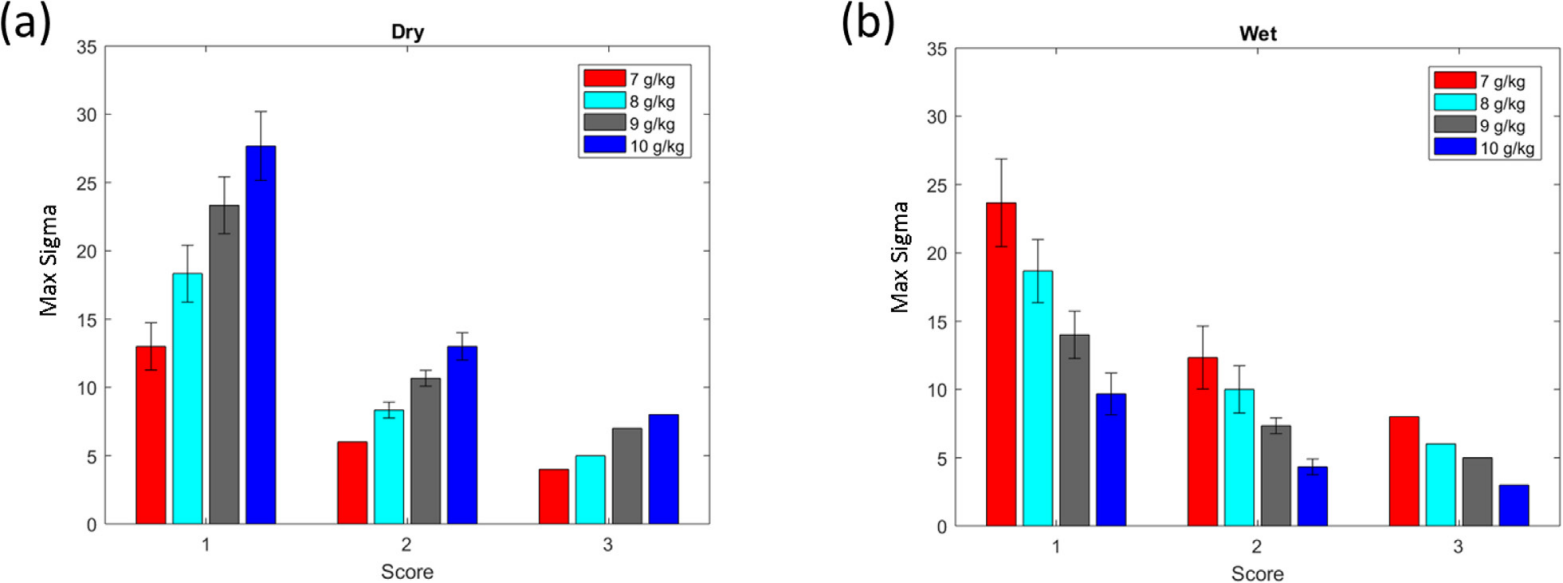
Results for scenario set III for product B using a humidity-dependent recipe. In this scenario, score indicates the number of parameters deviating from target. (a) Results for simulations with dry deviations. (b) Results for simulations with wet deviations. Bars associated with score 3 do not have error bars as they contain only one simulation each.

The next analysis was to see how large the deviation from target could be for six occurrences, lasting five minutes each (scenario set III). As can be seen in Fig. 17, simulations with only one parameter deviating are quite robust to changes from target. However, as the number of parameters increases, there is less room for deviation. At the worst case, where three parameters are deviating, the largest magnitude of deviation is three standard deviations from target, again considerably worse than in the case of the robust process.

**Fig. 18:**
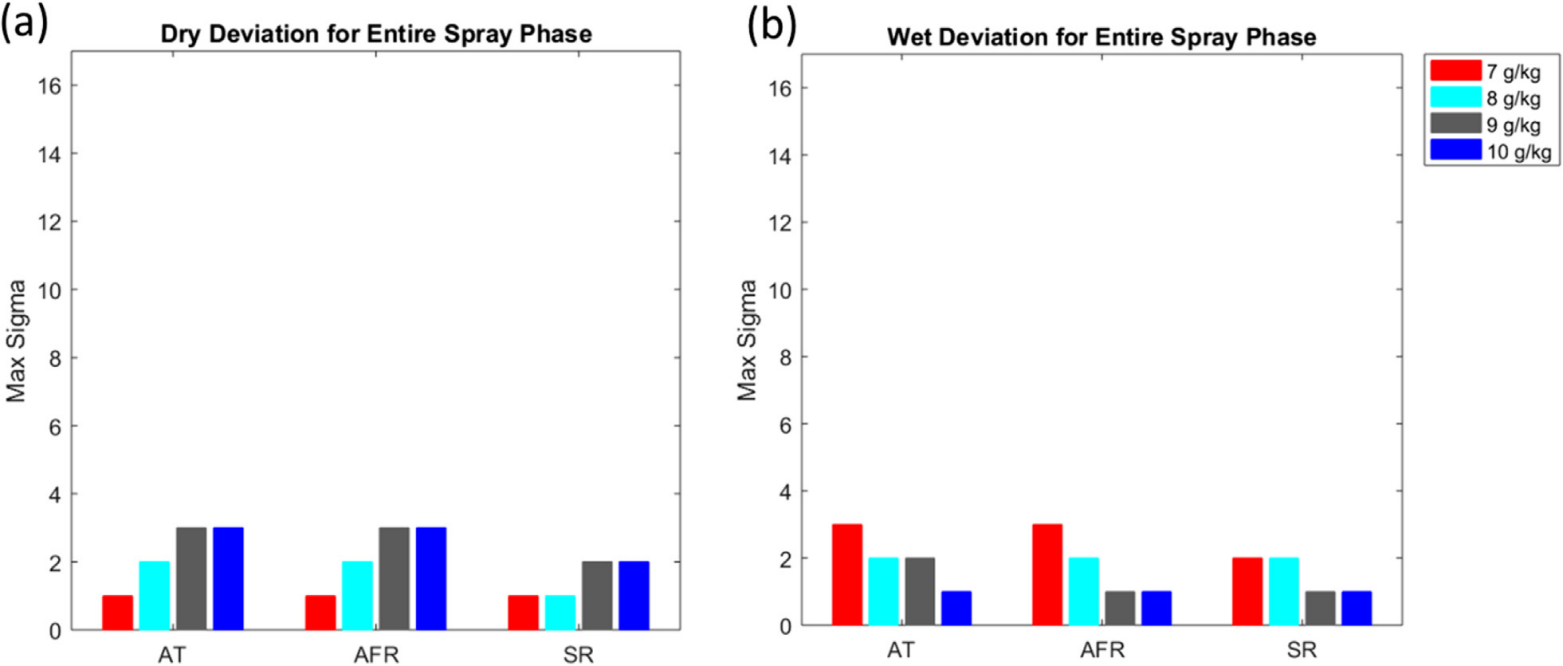
Results for scenario set IV for product B using a humidity-dependent recipe. (a) Results for simulations with dry deviations. (b) Results for simulations with wet deviations.

The results of the final analysis, scenario set IV, are presented in Fig. 18. There is very little room for deviations with this type of worst-case scenario, which implies accurate calibration of process monitoring sensors is a prerequisite for this product.

This case study has highlighted how a sensitive process may differ significantly from a robust one in terms of its “risk fingerprint”, generated by exploration of the various scenario sets. For this specific product, the presented findings prompted additional efforts to reduce the risk of maximum LOD excursions. Given that the overall robustness of the process is mediocre even in the case of humidity-dependent recipes, additional control approaches have been explored, including restriction of manufacturing of this product to mild periods, and feedback control using process analytical technology. The corresponding considerations involve aspects related to finance, planning, regulatory impact, etc. and are outside of the scope of this work.

### 4.3 Proven acceptable ranges (PARs)

As all four granulation process parameters are typically considered key and/or critical because of their impacts on tablet hardness and dissolution, their proven acceptable ranges (PAR) must be determined prior to process validation. The PAR is defined as “a characterised [*sic*] range of a process parameter for which operation within this range, while keeping other parameters constant, will result in producing a material meeting relevant quality criteria.” (International Conference on Harmonisation of Technical Requirements for Registration of Pharmaceuticals for Human Use, 2009). Clearly, the types of simulations described in Section 3.2.1 can be used to help construct the PAR for fluid bed granulation processes as they allow identifying conditions under which the process will deviate dramatically from the target.

**Fig. 19:**
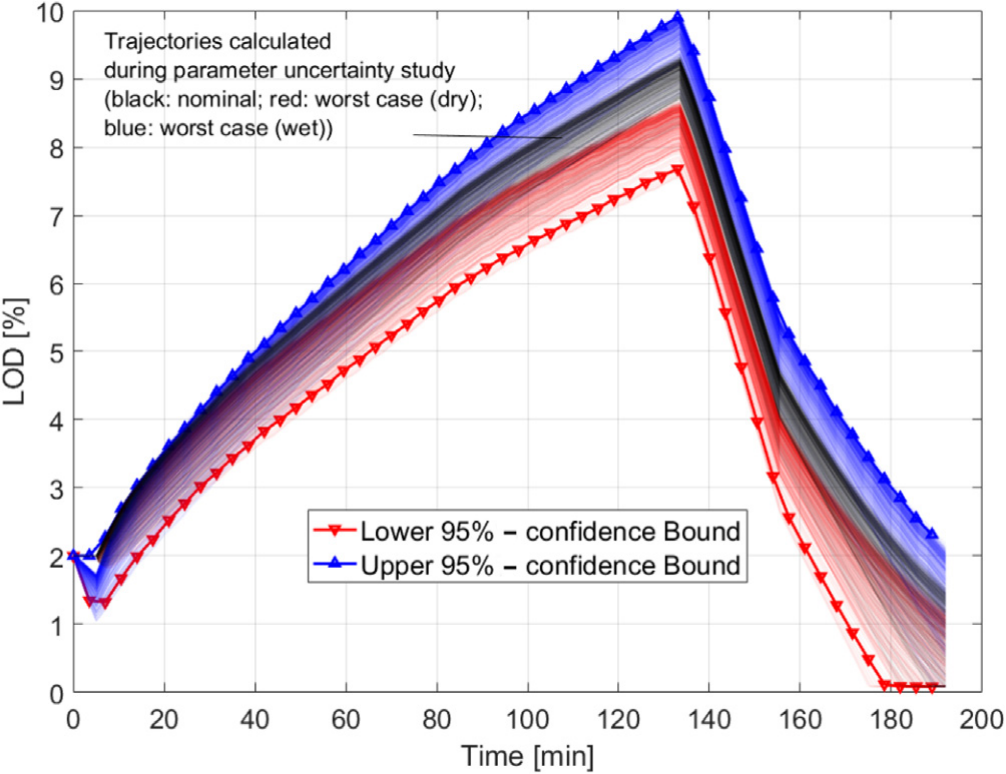
Overall uncertainty example (only spray rate disturbances are considered) for product C.

However, as discussed earlier, when defining explicit ranges, the effects of both the input process parameter variability and the model adjustable parameter uncertainty on the granulation are important. In order to ensure maximum robustness, it is crucial to not only have an understanding of the expected outcome of a process as predicted by some digital twin, but also its variability. For this reason, we have performed analyses of the total output uncertainty as outlined in Section 3.2.3 for selected products; an example result is given in Fig. 19.

While these assessments are of great value, note that for the determination of the PARs a holistic view and thus application of the entire process understanding is required. This includes factors outside of the scope of the model and which we have explicitly stated to be thought of as ‘under control’. For example, the PAR for the airflow additionally involves considerations with regards to the fluidization of the bed, while the PAR of the spray rate must take into account atomization pressure (ranges) and nozzle configurations. In our case, PARs were typically defined by the intersection of all found ranges in order to obtain the maximally robust process. The resulting ranges are of similar widths as those encountered in processes that were established using traditional, model-free approaches, yet are based on a much deeper physical understanding of the process.

## 5 Conclusion

In this work, we have provided insight into the application of a moisture model for fluid bed granulation during an industrial technology transfer in the pharmaceutical industry. To our knowledge, this is the first peer-reviewed publication that showcases the successful use of a predictive, mechanistic fluid bed granulation model for multiple pharmaceutical products across different pieces of equipment, and one of only a handful to tackle output uncertainty in this context. While only an excerpt of the results is shown, we believe the value added by using this tool to be plainly visible. First and foremost, the overall understanding of the process is dramatically increased. The LOD profile is used as the scale-independent parameter that links processes across scales, and ultimately this link is leveraged to draw conclusions for large scale from design space experiments at the small scale – at much reduced cost. Specifically, for this project, a conservative estimate would be that two commercial-scale batches did not have to be run for each of the five products, as all stakeholders were satisfied by the demonstrated understanding and control of the processes. In that regard, the importance of the pre-aligned and clearly defined acceptance criteria is to be emphasized. We leave it to the reader to ponder the associated savings in terms of material, energy, but also time resulting from reducing the number of large-scale batches.

Beyond this very tangible achievement, the uncertainty analyses outlined in this work demonstrate another advantage of using models: the ability to test myriads of scenarios quickly and at virtually no cost, allowing to identify potential problems much earlier and hence greatly increasing the overall robustness of each process. In the case at hand, these findings have directly led to assessments on how to avoid design space excursions for some products months before any equipment in the new plant was installed and qualified. This proactive approach is fully aligned with the quality-by-design framework and we believe it to be instrumental to guarantee the highest quality in our products.

Lastly, we hope this work demonstrates an important aspect encountered in pharmaceutical manufacturing of commercial products: pragmatism is key. Simplicity, verifiability, and transferability of a model trump completeness of the description almost every time. This holds not only for fluid bed granulation, but in fact for *all* mathematical process models. In other words, the business case for a complex model requiring substantial experimental effort to validate for any given product and/or operating range is only rarely positive, especially when that output is not directly and *quantifiably* linked to a critical quality attribute.

## Declaration of Competing Interest

The authors declare that they have no known competing financial interests or personal relationships that could have appeared to influence the work reported in this paper.

## References

[b0005] Chaterjee, S., et al., 2017. An Overview of the Role of Mathematical Models in Implementation of Quality by Design Paradigm for Drug Development and Manufacture, Food and Drug Administration Papers, pp. 23.

[b0010] Chaudhury A. (2013). Multi-dimensional mechanistic modeling of fluid bed granulation processes: an integrated approach. Adv. Powder Technol..

[b0015] de Albuquerque I. (2016). Effect of needle-like crystal shape on measured particle size distributions. AIChE J..

[b0020] Djuris J. (2017). Modeling in the quality by design environment: regulatory requirements and recommendations for design space and control strategy appointment. Int. J. Pharm..

[b0025] FDA (2012). Guidance for Industry, Q8, Q9, & Q10 Questions and Answers, Appendix Q&As from Training Sessions, U.S. Department of Health and Human Services, Food and Drug Administration (FDA), Center for Drug Evaluation and Research (CDER) & Center for Biologics Evaluation and Research (CBER).

[b0030] Gagnon F. (2017). Nonlinear model predictive control of a batch fluidized bed drzer for pharmaceutical particles. Control Eng. Pract..

[b0035] Garcia Munoz S. (2010). Handling uncertainty in the establishment of a design space for the manufacture of a pharmaceutical product. Comput. Chem. Eng..

[b0040] Garcia Munoz S. (2018). A flowsheet model for the development of a continuous process for pharmaceutical tablets: an industrial perspective. AIChE J..

[b0045] Gupta R. (2017). Fluid bed granulation and drying. Predictive Modeling of Pharmaceutical Unit Operations.

[b0050] Heinrich S. (2005). Fluidized bed spray granulation: analysis of heat and mass transfers and dynamic particle populations. Braz. J. Chem. Eng..

[b0055] Hu X. (2008). Understanding and predicting bed humidity in fluidized bed granulation. J. Pharm. Sci..

[b0060] International Conference on Harmonisation of Technical Requirements for Registration of Pharmaceuticals for Human Use (2009). Pharmaceutical Development Q8(R2). 10.1111/j.1365-2125.1994.tb05705.x PMC1364893 8054244

[b0065] Korakianiti E. (2011). Statistical thinking and knowledge management for quality-driven design and manufacturing in pharmaceuticals. Pharm. Res..

[b0070] Lyngberg O., Ierapetritou M.G., Ramachandran R. (2016). Applications of modeling in oral solid dosage form development and manufacturing. Process Simulation and Data Modeling in Solid Oral Drug Development and Manufacture.

[b0075] Mortier S.T.F.C. (2011). Mechanistic modelling of fluidized bed drying processes of wet porous granules: a review. Eur. J. Pharm. Biopharm..

[b0080] Muddu S.V. (2018). Model development and validation of fluid bed wet Granulation with dry binder addition using a population balance model methodology. Processes.

[b0085] Ochsenbein D.R. (2015). Agglomeration of needle-like crystals in suspension. II. Modeling. Cryst. Growth Des..

[b0090] Pauli V. (2019). Orthogonal redundant monitoring of a new continuous fluid-bed dryer for pharmaceutical processing by means of mass and energy balance calculations and spectroscopic techniques. J. Pharm. Sci..

[b0095] Rambali B. (2001). Using experimental design to optimize the process parameters in fluidized bed granulation on a semi-full scale. Int. J. Pharm..

[b0100] Sen M. (2014). A multi-scale hybrid CFD-DEM-PBM description of a fluid-bed granulation process. Processes.

[b0105] Van Bockstal P.-J. (2017). Quantitative risk assessment via uncertainty analysis in combination with error propagation for the determination of the dynamic Design Space of the primary drying step during freeze-drying. Eur. J. Pharm. Biopharm..

[b0110] Vetter T. (2015). Designing robust crystallization processes in the presence of parameter uncertainty using attainable regions. Ind. Eng. Chem. Res..

