# Supplementary material for: Industrial application of heat- and mass balance model for fluid-bed granulation for technology transfer and design space exploration
Source: Int J Pharm X. 2019 Aug 12;1:100028. doi: 10.1016/j.ijpx.2019.100028 (PMC6733368; doi:10.1016/j.ijpx.2019.100028)
Supplement: Supplementary data 1 [file mmc1.docx]

**Supplementary Material for Industrial Application of Heat- and Mass Balance Model for Fluid-bed Granulation for Technology Transfer and Design Space Exploration**

*David R. Ochsenbein, Matthew Billups, Bingbing Hong, Elisabeth Schäfer, Alexander J. Marchut, and Olav K. Lyngberg*

**Additional equations for mass- and heat balance model**

Energy transfer due to air flow in:

(A1)

$$\begin{aligned} \dot{H}_{\mathrm{air}}^{\mathrm{in}}= F_{\mathrm{air}}\left( c_{p,a}\left( T_{\mathrm{in}} - T_{0} \right)+ x_{\mathrm{in}}\left( \Delta H_{\mathrm{evap}} + c_{p,w}^{g}\left( T_{\mathrm{in}} - T_{0} \right) \right) \right) \end{aligned}$$

Here, $T_{\mathrm{in}}$ corresponds to the inlet air temperature, while $\Delta H_{\mathrm{evap}}$ is the evaporation enthalpy.

Analogously, the energy transfer due to air flow out is given by:

(A2)

$$\begin{aligned} \dot{H}_{\mathrm{air}}^{\mathrm{out}}= F_{\mathrm{air}} \left( c_{p,a}\left( T_{\mathrm{air}} - T_{0} \right)+ x\left( \Delta H_{\mathrm{evap}} +c_{p,w}^{g}\left( T_{\mathrm{air}} - T_{0} \right) \right) \right) \end{aligned}$$

The energy transfer due to the spray is:

(A3)

$$\begin{aligned} \dot{H}_{\mathrm{spray}}= F_{\mathrm{spray}}\left( w_{s} c_{p,b} + \left( 1 - w_{s} \right)c_{p,w}^{\mathcal{l}} \right) \left( T_{\mathrm{spray}}- T_{0} \right) \end{aligned}$$

While the energy transfer due to evaporation is modeled by:

(A4)

$$\begin{aligned} \dot{H}_{\mathrm{evap}}= \dot{m}_{\mathrm{evap}}\left( \Delta H_{\mathrm{evap}}+ c_{p,w}^{g}\left( T_{\mathrm{bed}}-T_{0} \right) \right) \end{aligned}$$

The heat transfer from bed to vapor is

(A5)

$$\begin{aligned} \dot{Q}_{bed,air}=\beta_{bed,air} A_{p}\left( \theta_{p} \right)\left( T_{\mathrm{bed}} - T_{\mathrm{air}} \right) \end{aligned}$$

where the variable $\beta$ is used to indicate a heat transfer coefficient estimated from literature correlations, and $A_{p}\left( \theta_{p} \right)$ is the particle surface area, the only other item that is a function of one of the model parameters.

Similarly, the heat transfer from bed to wall is

(A6)

$$\begin{aligned} \dot{Q}_{bed,wall}= \beta_{bed,wall} A_{\mathrm{bed}} \left( T_{\mathrm{bed}} - T_{\mathrm{wall}} \right) \end{aligned}$$

while that from wall to environment is:

(A7)

$$\begin{aligned} \dot{Q}_{wall, env}= \beta_{wall, env} A_{\mathrm{outer}} \left( T_{\mathrm{wall}}-T_{\mathrm{env}} \right) \end{aligned}$$

and that from air to wall is.

(A8)

$$\begin{aligned} \dot{Q}_{air,wall}=\beta_{air, wall} A_{\mathrm{inner}} \left( T_{\mathrm{air}}- T_{\mathrm{wall}} \right) \end{aligned}$$

In Equations A5 to A8, the terms $A_{i}$ are surface areas calculated either from known equipment dimensions (and the approximated bed height).
